# Protons as Messengers of Intercellular Communication in the Nervous System

**DOI:** 10.3389/fncel.2018.00342

**Published:** 2018-10-10

**Authors:** Enrique Soto, Audrey Ortega-Ramírez, Rosario Vega

**Affiliations:** Instituto de Fisiología, Benemérita Universidad Autónoma de Puebla, Puebla, Mexico

**Keywords:** ASIC, vestibule labyrinth, cochlea, amygdala, fear

## Abstract

In this review, evidence demonstrating that protons (H^+^) constitute a complex, regulated intercellular signaling mechanisms are presented. Given that pH is a strictly regulated variable in multicellular organisms, localized extracellular pH changes may constitute significant signals of cellular processes that occur in a cell or a group of cells. Several studies have demonstrated that the low pH of synaptic vesicles implies that neurotransmitter release is always accompanied by the co-release of H^+^ into the synaptic cleft, leading to transient extracellular pH shifts. Also, evidence has accumulated indicating that extracellular H^+^ concentration regulation is complex and implies a source of protons in a network of transporters, ion exchangers, and buffer capacity of the media that may finally establish the extracellular proton concentration. The activation of membrane transporters, increased production of CO_2_ and of metabolites, such as lactate, produce significant extracellular pH shifts in nano- and micro-domains in the central nervous system (CNS), constituting a reliable signal for intercellular communication. The acid sensing ion channels (ASIC) function as specific signal sensors of proton signaling mechanism, detecting subtle variations of extracellular H^+^ in a range varying from pH 5 to 8. The main question in relation to this signaling system is whether it is only synaptically restricted, or a volume modulator of neuron excitability. This signaling system may have evolved from a metabolic activity detection mechanism to a highly localized extracellular proton dependent communication mechanism. In this study, evidence showing the mechanisms of regulation of extracellular pH shifts and of the ASICs and its function in modulating the excitability in various systems is reviewed, including data and its role in synaptic neurotransmission, volume transmission and even segregated neurotransmission, leading to a reliable extracellular signaling mechanism.

## Introduction

We first review evidence related to extracellular proton production and of its regulation and control mechanisms, then the structure and functions of the Acid Sensing Ion Channels (ASIC) are discussed, along with other extracellular pH sensing molecules, and finally evidence of the role of proton signaling mechanism in specific synaptic transmission, volume neurotransmission, and segregated specific proton production are reviewed.

Extracellular pH is a highly controlled variable, with many regulatory processes maintaining it within a restricted value, which in vertebrates is close to neutrality. The proper function of organisms very much depends on pH homeostasis and at the systems level, its variations are minimal, otherwise catastrophic failure of organism occurs. The highly controlled extracellular H^+^ concentration allows for its small local variations to be “read” as specific signals. Somehow during evolution, cells develop mechanisms for the sensing of pH variations produced by activity in its neighboring cells. The driving force for evolution of these mechanisms most likely takes place during the transition to multicellular organisms, thus constituting a signaling system. Proton signaling is very much like the Ca^2+^ signaling mechanisms (Carafoli, 2005), but mainly act on extracellular mediums. Evidence has accumulated showing that H^+^ may accumulate in nano- or micro-domains and that they may operate as intercellular messengers (Beg et al., 2008). In higher organisms, proton-mediated signaling has been found to work in concert with classical neurotransmission, mediating various processes such as fear conditioned learning, retinal cell activation, inhibition of convulsive crisis, and in transduction and sensory coding in various systems (Li and Xu, 2011).

Buildup of extracellular proton concentration may be the consequence of metabolic activity, thus releasing protons in a constitutively unregulated form, or H^+^, may also be co-released with classical neurotransmitters in a regulated form. Extracellular H^+^ concentration increase may also be produced by a specific transport mechanism acting together with the buffer capacity of the media. There is no evidence of regulated independent H^+^ release in any synapse. However, it has been found that protons fulfill most of the criteria in order to be considered a neurotransmitter; including release in the synapse, postsynaptic receptors, mechanisms to remove them from synaptic cleft, exogenous application resembling normal system activation, agonists resembling normal activation of the system, and antagonists possibly blocking the postsynaptic response (Du et al., 2017). A problem of classifying protons as neurotransmitters is related to the fact that its regulated release is always a co-release with classical neurotransmitters, which results as a byproduct of neurotransmitter transport mechanisms into the synaptic vesicles. However, regulated extracellular medium acidification by means of the activation of transporters and exchange molecules may lead to a very restricted proton accumulation. Therefore, it seems appropriate to consider the system formed by H^+^ as a messenger and its specific receptors the ASIC as an extracellular signaling mechanism, which may modulate various neuronal processes, and have a salient role in the pathophysiology of various diseases of the central nervous system (CNS). Some of these processes, which we have reviewed, are related to metabolic buildup of extracellular proton concentration; the fear response in the amygdala, the inhibitory neuron activation in convulsive crisis and the motor response in hypoxia, among others. In these cases, a mass of neurons leads to an increase in extracellular H^+^ concentration, which activates ASICs, expressed either at the synaptic or extrasynaptic level. The extrasynaptic activation of receptors has been shown for various neurotransmitters, such as dopamine, serotonin and cannabinoids (Del-Bel and De-Miguel, 2018), leading to the concept of volume transmission, which is a form of communication mediated by extracellular diffusion of transmitter substances through extracellular space (Fuxe et al., 2007).

Restricted actions of H^+^ have been shown in the lateral amygdala (Du et al., 2014) and in the nucleus accumbens, where protons contribute to the excitatory postsynaptic current (EPSC) (Kreple et al., 2014). To define the extent at which proton concentration in synaptic like nano-domains may activate the ASICs, a construct in HEK293T cells was devised, showing that H^+^ current passing through light activated *Archaerodopsin-3* or voltage-gated proton channel (Hv1) can activate closely coupled ASIC channels and induce its activation in closely located “sniffer” cells (Zeng et al., 2015). Modeling of this system showed that proton currents may lead to a pH change of almost 4 units (from 7.4 to 4 in a solution with 10 mM HEPES) and of 0.6 (from 7.4 to 6.8 units in a solution with 22 mM NaHCO_3_) within 10–100 nm (Zeng et al., 2015). These results demonstrate that extracellular H^+^ concentration changes in nano-domains can activate the ASICs. Thus, evidence for restricted signaling by protons is feasible and evidence has been obtained both from native and heterologous expression systems.

## Proton Homeostasis and Proton Accumulation

Over time, organisms have had to adapt to diverse environments, redefining their characteristics; such as cellular pH regulation, cell volume, and maintaining ionic homeostasis to survive, reproduce and preserve their species, some of which have been able to evolve in extreme conditions up to the present (Rothschild and Mancinelli, 2001). Although environmental variables are critical for the evolution of species, it is known that the regulation of extracellular pH (pHe) and intracellular pH (pHi) is essential for life, since it is related to enzymatic processes, ionic modulation, and nutrient homeostasis. There is also evidence that the structuring of the genetic code occurred in an acidic environment, therefore, a large spectrum of membrane proteins with highly specialized functions for the preservation of cellular pH homeostasis have emerged during evolution (Brett et al., 2005; Di Giulio, 2005; Daniel et al., 2006).

To maintain the pHe within the physiological limits (7.3–7.4 in higher vertebrates) (Casey et al., 2010), there must be a balance between the contribution of the production of H^+^ and the buffering or elimination of H^+^. The mobile buffer systems that regulate pHe in the CNS include the bicarbonate/carbonic acid system (HCO_3_^-^/H_2_CO_3_), hemoglobin, plasma proteins and phosphates, of which the most significant is the HCO_3_^-^/H_2_CO_3_ (about 75% of the total of buffer capacity of the blood) (Chesler, 2003). In addition to the mobile buffer systems, there are several cytoplasmic transporters that carry protons through the membrane in order to maintain pHi (pH 7.2) and pHe values within physiological limits (Casey et al., 2010). Metabolic reactions, protein catabolism and organic acids can produce intense intracellular acidification, which is why most H^+^ transporters are responsible for alkalizing the cytosol, extruding protons, or capturing them in intracellular vesicles and organelles. The H^+^ transporters present in vertebrate cells include: Na^+^/H^+^ exchangers (NHE), HCO_3_^-^ transporters, Vacuolar H^+^-ATPase (V-ATPase), monocarboxylic acid transporters and the carbonic anhydrase enzyme family (CAs), among others (**Table 1**; Obara et al., 2008 for reviews see: Chesler, 2003; Verma et al., 2015; Zhao et al., 2016).

**Table 1:** Transporters and enzymes involved in H^+^ extrusion and loading in the CNS.

Transporter		Isoforms	Distribution	Function	Reference
Na^+^/H^+^ exchanger		NHE1-NHE9	All are expressed in mammalian CNS cells. NHE1-NHE5 plasmalemmal localization, NHE6 y NHE7 intracellular localization	NHE catalyzes the exchange of one extracellular sodium ion for one intracellular proton	Slepkov et al., 2007; Donowitz et al., 2013
Bicarbonate transporters		
	Na^+^/HCO3^-^ cotransporters	NBCe1-2, NBCn1-2, NDCBE	Brain, choroid plexus, and meninges	Mediate cotransport of Na^+^ and base (HCO_3_^-^ and/or CO_3_^2-^) may act as acid extruder or loaders	Obara et al., 2008; Parker and Boron, 2013
	Anion exchangers	AE1-AE4	Brain, retina, salivary glands, mature erythrocytes, and immature cultured oligodendrocytes	Generally act as acid loaders, extruding HCO_3_^-^ in exchange for Cl^-^ influx.	Obara et al., 2008; Zhao et al., 2016
	Na^+^-driven Cl^-^/HCO3^-^ exchanger	NCBE, NDCBE	Cerebral cortex, cerebellum, medulla, thalamus, hippocampus	Removes extracellular Na^+^ in exchange for intracellular Cl^-^. This process is associated with HCO_3_^-^ influx and H^+^ efflux.	Sinning and Hübner, 2013; Zhao et al., 2016
Vacuolar type proton ATPase		V-ATPase	Astrocytes and neurons	Using the ATP hydrolysis derived energy, transports protons from cytoplasm into either the lumen of single membrane organelles, or extracellular space	Obara et al., 2008; Casey et al., 2010
Monocarboxylic acid transporters		MCT1–MCT4	Blood vessels, astrocytes, neurons	Cotransport of one monocarboxylate anion (lactate, pyruvate, acetoacetate and/or b-hydroxybutyrate) with one proton	Obara et al., 2008
Carbonic anhydrases		CA I, II, III, VII, XIII, IV, IX, XII, XIV, XV, and VI	Nervous tissue of different species of mammals (intra and/or extracellular location)	Catalyze the inter conversion of CO_2_ and H_2_O and the dissociated ions of carbonic acid (i.e., bicarbonate and protons)	Obara et al., 2008: Sinning and Hübner, 2013


Homeostasis of pHi and pHe is crucial in the CNS, since it is related to neuronal excitability and neurotransmission. In the brain, neuronal activity causes local and transient pH changes during physiological processes; neuronal activity can induce a transient and localized pH fluctuation at synaptic cleft that varies from 0.2 to 0.6 units, depending on stimulation protocol (Zeng et al., 2015). The loading of neurotransmitters in the synaptic vesicles occurs due to the action of the V-ATPase, so the synaptic vesicles have an acidic pH (pH Δ 5.2–5.7) (Storozhuk et al., 2016). During neurotransmission, vesicular content is released into synaptic space, co-releasing neurotransmitters and protons, and thus producing a brief but intense acidification followed by a slow alkalization (Sinning and Hübner, 2013).

**FIGURE 1:**
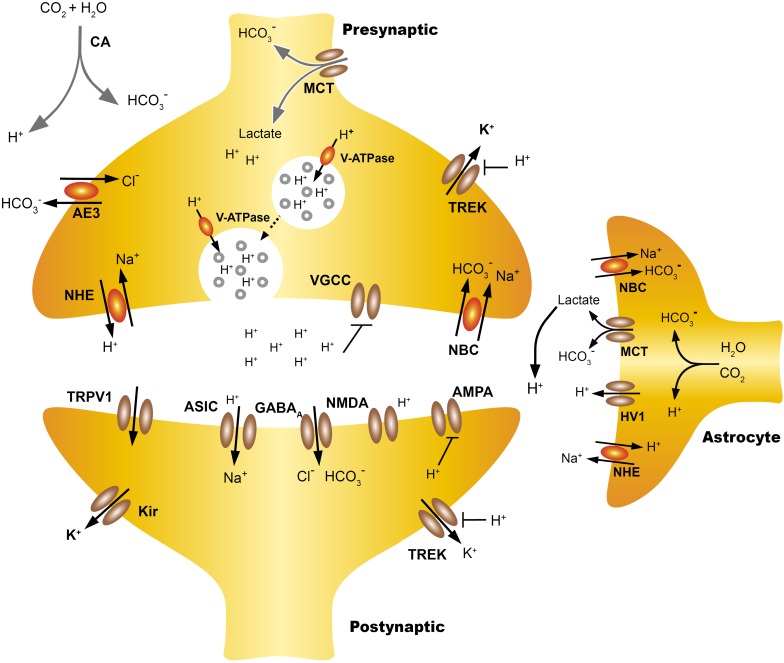
Extracellular proton homeostasis. Presynaptic cells express pumps and transporters that contribute to control pHi, including monocarboxylate transporters (MCT), anion exchanger (AE), Na^+^ -H^+^ exchangers (NHE), coupled sodium bicarbonate transporters (NBC/NCBTs) and the V-ATPase. During neurotransmission, the extracellular acidification of the synaptic cleft can take place with both rapid or slow kinetics. Synaptic vesicles co-release protons and neurotransmitters, also the transient incorporation of V-ATPase in the plasma membrane contribute to the extrusion of protons, producing rapid acidification of the synaptic cleft. Also intensive activity increases the energy demand of astrocytes, which increases the production of lactate and CO_2_, this can diffuse freely while the lactate is transported from the astrocyte to the extracellular space by MCT, which leads to a slow extracellular acidification. Lactate may also be taken from the presynaptic neuron as a source of energy. TWIK-related K^+^ channels (TREK) are modulated both by pHe and pHi, the intracellular H^+^ increase the open probability of TREK-1, hyperpolarizing the cells while extracellular H^+^ inhibits TREK-1 and also voltage gated calcium channels (VGCC). The NBC transporters are widely expressed in neurons and the astrocytes and their action by introducing HCO_3_ from the extracellular mediums, producing gradual extracellular acidification. Acid pHe can also activate postsynaptic channels like ASIC or TRPV1 while reducing the open probability of NMDAR. The regulation and actions of pHi and pHe at the synapse are discussed in more detail in Proton Homeostasis and Proton Accumulation.

In neurons, the main acid is the AE3 chloride-bicarbonate exchanger, an electroneutral exchanger that extrudes one HCO3^-^ by one Cl^-^ (Ruffin et al., 2014). The main acid extruders are the Na^+^-H^+^ exchangers (mainly NHE1, NHE3, and NHE5 in the CNS) which exchange one Na^+^ for one H^+^ (Donowitz et al., 2013) and the Na^+^-coupled HCO_3_- transporters (NCBTs) (Parker and Boron, 2013). Usually NHEs exchange an intracellular H^+^ for an extracellular Na^+^ (dissipating the inward gradient for Na^+^), restoring pHi after an acid load. The Na^+^ -dependent exchangers (Na^+^-driven Cl^-^/HCO_3_^-^) are highly expressed in the cerebellum, cerebral cortex, thalamus and hippocampus, they remove intracellular Cl^-^ in exchange for extracellular Na^+^ and HCO_3_^-^, a process which implies bicarbonate influx and proton efflux (Alvadia et al., 2017). In mice the disruption of Na^+^-driven Cl-/HCO3- increased the seizure threshold (Sinning and Hübner, 2013; Zhao et al., 2016; **Figure 1**).

The transport systems have a primarily homeostatic function related to the maintenance of intracellular pH. Its role in proton signaling processes depends on their colocalization with pH-sensitive ion channels in membrane micro-regions. Therefore, in the study of the expression of transporters, it is essential to define its location in membrane micro-regions. For example, action potential firing in cultured hippocampal neurons induces the activation of glutamate N-methyl-D-aspartate receptors (NMDA_R_) that may recruit NHE5 to the dendritic membrane surface, where it will contribute to synaptic cleft acidification and suppression of dendritic spine growth (Diering et al., 2011). The knock-down of NHE5, or overexpression of a dominant-negative mutant, causes dendritic spine overgrowth (Diering and Numata, 2014). Of most interest was the recent demonstration that links the NHE9 coding gen -Slc9a9- to autism spectrum disorders (ASD). The elimination of NHE9 in mice produced an ASD-like behavior and provides the field with a new mouse model of ASDs (Yang et al., 2016).

In relation to the role of H^+^ as synaptic co-transmitters, the V-ATPase plays a pivotal role in transporting protons from the cytoplasm into synaptic vesicles using energy from ATP hydrolysis. Also, synaptic vesicle fusion during neurotransmitter release transiently incorporates V-ATPase into the synaptic membrane, where it contributes to the acidification of synaptic cleft (Casey et al., 2010). Regardless of the neurotransmitter, synaptic vesicles (SV) express the V-ATPase, its activity produces a concentration gradient of H^+^ (ΔpH), and an electrical potential (Δψ) in the membrane of the SV. The electrochemical gradient (ΔμH^+^) is used by the vesicle transporters to charge neurotransmitters in the SV. Although most neurotransmitters use V-ATPase derived gradients, there are differences in intravesicular H^+^ concentrations and transport mechanisms. For example, glutamatergic SV exhibit higher acid luminal vesicular pH (pH ∼5.8) than GABAergic SV (∼6.4) (Eriksen et al., 2016; Farsi et al., 2016). The loading of glutamate depends on the vesicular glutamate transporters (VGLUT), which function as a glutamate/proton exchanger associated with a channel-like chloride conductance (Martineau et al., 2017). The Cl^-^ conductance accounts for the Cl^-^ dependence of VGLUT activity (Egashira et al., 2016; Martineau et al., 2017). In contrast, Gamma aminobutyric acid (GABA) loading in SV is done by vesicular GABA transporters (VGAT), these require ΔμH^+^ for optimal activity. VGAT operates as a GABA/H^+^ antiporter, with no other ions participating in the transport (Farsi et al., 2016).

## ASICs

The ASICs are chemically gated ion channels that are voltage-insensitive, cation-selective, (mostly permeable to Na^+^) and non-specifically blocked by amiloride, they belong to an evolutionary old channel family, the Epithelial Sodium Channel/Degenerins (ENac/DEG) (Krishtal and Pidoplichko, 1980; Krishtal and Pidoplichko, 1981; Waldmann et al., 1997; Kellenberger and Schild, 2002; nicely reviewed by Hanukoglu, 2017). There are at least seven isoforms of the ASICs (1a, 1b, 2a, 2b, 3, 4,and 5) derived from five *ACCN1-5* genes (HUGO Gene Nomenclature Committee). They are widely expressed in the peripheral and central nervous system as well as other tissues. Different studies have shown that activation of these channels is linked to various physiological processes, such as pain sensing, auditory and visual processing, fear conditioning, drug addiction, epilepsy ending, and in pathological processes such as anxiety, ischemia and multiple sclerosis (Pignataro et al., 2007; Xiong et al., 2008; Wemmie et al., 2013).

**FIGURE 2:**
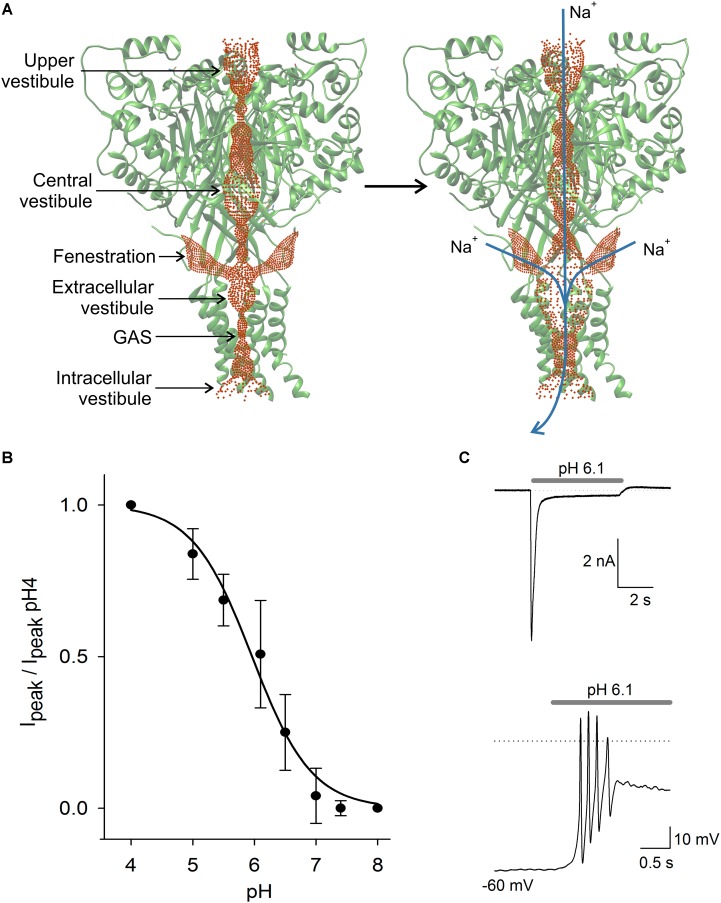
ASIC structure and properties. In **(A)** scheme of the ASIC channel trimer in the closed and open states. Current is activated by H^+^ and carried by Na^+^ and in lower proportion by Ca^2+^. Activation of the ASIC led to a significant expansion of the central pore, due to a complex modification of the channel structure. The three lateral fenestrations would significantly contribute to ion passage into the extracellular vestibule. In **(B)** pH dependence of ASIC activation in DRG neurons. The current showed typical sigmoidal pH dependence with a pH_50_ of 6.1. In **(C)** typical ASIC current in voltage clamp from a DRG neuron produced by pH 6.1 solution perfusion. Current reached a peak and then desensitized during the first second to a plateau of sustained current. In the lower panel in current clamp condition, the perfusion of pH 6.1 to a DRG neuron induced a series of action potentials followed by a large sustained depolarization, coinciding with the recording in voltage clamp.

Typically, ASIC currents show a peak current followed by complete or partial desensitization, depending on the subunit composition of the channel (Hesselager et al., 2004**).** Functional ASICs are formed by trimeric proteins. Studies of homomeric channels show that ASIC isoforms have important differences in the affinity for protons, ASIC3 is the most sensitive unit with a pH_50_ of 6.4, and the least sensitive is the ASIC2a with a pH_50_ of 4.5. (ASIC1b = 6.1; ASIC 1a 5.8 and the ASIC4 and ASIC2b did not form functional homomeric channels) (**Table 2**). Thus, functionally, the ASICs span about four units range of pH sensitivity (from about 4 to 8). The ASIC subunits also differ in their current kinetics. ASICs activate within <5 ms in the ASIC3 to 6–14 ms for the ASIC2a (Bässler et al., 2001; Li et al., 2010), and the current desensitizes with variable kinetics. The desensitization and inactivation coefficient (ratio of the current at the end of desensitization versus peak current) in the continual presence of protons significantly defines the functional consequences of ASIC activation (**Figure 2**). The ASIC1a and 1b current almost completely desensitizes, while the ASIC2a slowly desensitizes and ASIC3 quickly desensitizes, although a sustained component is exhibited, which is always about 0.3 of the peak current (Gründer and Pusch, 2015). Thus, the total current carried by ASIC3 is much larger than that of ASIC1, and its activation in neurons induces a sustained depolarization and large Na^+^ inflow. The properties of heteromers are unpredictable from those of homomers. In the CNS, the ASIC1a seems to be the most prominently expressed of the ASICs, although ASIC2a and ASIC2b are also significantly expressed in some regions of the brain, and ASIC3 and ASIC4 have a restricted expression (Wu J. et al., 2016). ASIC2a and ASIC2b interact with ASIC1a to form heteromeric channels, shifting the pH sensitivity and desensitization kinetics of acid gated currents (Askwith et al., 2004; Hesselager et al., 2004). ASIC2a interacts with PSD 95 protein and was shown to contribute to the transport of ASIC1a containing channels in dendritic spines (Zha et al., 2009). Although the desensitization process of ASICs casts doubt on their potential to follow rapid neuronal signaling, there is evidence that activation by small pH changes produced practically no desensitization and recovery seems to be very fast in relation to neuronal activity (MacLean and Jayaraman, 2016). An excellent study of the ASIC response to rapid pH changes showed that currents from ASIC1a homomers and ASIC1a/2a heteromers may deactivate with very fast time constants (1a ≅ 0.7 – 1a/2a ≅ 0.3 ms), and that unusually slow desensitization rate (1a ≅ 700 – 1a/2a ≅ 780 ms) endows these receptor channels with the capability of following fast trains of stimuli during long lasting periods (1ms at 50 Hz during 2 s), suggesting that they may sustain postsynaptic responses when other receptors desensitize (MacLean and Jayaraman, 2016). In native ASICs in the dorsal root ganglion (DRG), neuron deactivation was ≈ 0.33 ms (MacLean and Jayaraman, 2016), although currents showed a higher variability of their desensitization kinetics due to the expression of ASIC1a, ASIC2a, and ASIC3 heteromeric channels (Kusama et al., 2013). Moreover, recovery from desensitization and deactivation kinetics of ASICs was dependent on pH, with a significant reduction of kinetics with acidic pH. Deactivation of the ASIC1a/2a changes from an extremely fast <1 ms deactivation at pH 8 to a slow >300 ms deactivation at pH 7.0 (MacLean and Jayaraman, 2017). This implies that charge transfer in ASICs will be dependent on the extracellular pH, which makes ASICs very unique among ligand-gated channels.

**Table 2:** Subunits, distribution and functions of ASICs.

Gene	Subunit	pH_50_	Distribution	Physiology	Reference
*ACCN2*	ASIC1a	5.8	CNS/PNS	Synaptic plasticity, learning and memory, fear conditioning, visual transduction, visceral mechano-reception, primary muscle hyperalgesia, apoptosis, chondroprotection and bone resorption	Wemmie et al., 2003; Hesselager et al., 2004; Ettaiche et al., 2006; Weng et al., 2007; Walder et al., 2011; Li et al., 2014
	ASIC1b	6.1	PNS		Gründer et al., 2000; Hesselager et al., 2004; Ugawa et al., 2006
*ACCN1*	ASIC2a	4.5	CNS/PNS	Visual transduction, detection of sour taste, mechanosensation, arterial baroreceptor reflex	Hesselager et al., 2004; Ettaiche et al., 2006; Ortega-Ramírez et al., 2017.
	ASIC2b	NA	CNS/PNS	Integrity of retina, modulator of ASIC1a, ASIC1b, ASIC2a, and ASIC3 currents	Ettaiche et al., 2006; Sherwood et al., 2011
*ACCN3*	ASIC3	6.4	CNS/PNS	Chemoreception], skin mechanosensory, auditory and visual processing, mechanosensory of the intestinal tract	Hesselager et al., 2004; Ettaiche et al., 2009; Lin et al., 2016; Ortega-Ramírez et al., 2017
*ACCN4*	ASIC4	NA	CNS/PNS	Modulate the amount of functional ASICs into the plasma membrane and as a regulator of pain	Donier et al., 2008


*ACCN, Amiloride-Sensitive Cation Channel; CNS: Central Nervous System; PNS, Peripheral Nervous System; pH50, Half-maximum pH of activation.*

Among the ASICs, the ASIC5 is the lesser known. It is phylogenetically between the ASIC and the ENaCs, although it has a 30% homology with ASICs, it is not activated by protons but by bile acids, leading to the denomination of BASIC (Lefèvre et al., 2014). It was shown that channel activates by membrane-active substances, suggesting that BASIC is sensitive to changes in the membrane structure (Schmidt et al., 2014). In the brain, ASIC5 is restrictively expressed in interneurons in the granular layer in a subset of Unipolar Brush Cells of the ventral uvula and nodulus in the cerebellum, where its specific function is thought to be critical and distinctive (Boiko et al., 2014).

There are also important pharmacological differences between the ASICs (Osmakov et al., 2014). The ASIC1a is particularly sensitive to Hi1a and PcTx1 toxins, the ASIC3 is blocked by APETx2, while ASIC2 is positively modulated by MitTx and by ApTx2 (Cristofori-Armstrong and Rash, 2017). The PcTx is mainly inhibitory in ASIC1a while it seems to potentiate the current in ASIC1b homomers and ASIC1a/ASIC2a heteromeric channels in a state-dependent manner. This action was also shown in ASIC currents recorded in rat cortical neurons (Chen et al., 2006; Liu et al., 2018). Thus, pharmacological specificity contributes to defining the role of specific subunits in certain CNS processes (Wu J. et al., 2016). The desensitization rate and tachyphylaxis are modulated by extracellular anions. Extracellular Cl^-^ slowed desensitization and increased tachyphylaxis in a dose-dependent form both in native hippocampal ASICs and in transfected ASIC1a channels (Kusama et al., 2010); in ASIC2a and ASIC3, anions also modulate the kinetics of desensitization and the pH dependence of the activation (Kusama et al., 2013). Interestingly, it has been shown that ASIC currents may be modulated by GABA(A) receptor currents in hippocampal neurons in DRG neurons and in HEK293 expression cells, suggesting that these two ion channels are within a microdomain where they may functionally interact. GABA receptors (GABA_R_) activation decrease the response of ASICs to pHe changes and ASIC1a activation also modifies the kinetics of GABA(A) receptor (Chen et al., 2011; Zhao et al., 2014). Also, other endogenous molecules such as spermine, agmatine, arachidonic acid, serotonin, dynorphins, and histamine may modulate the ASICs (Wemmie et al., 2013; Nagaeva et al., 2016; Wang et al., 2016; Ortega-Ramírez et al., 2017).

The ASIC subunits are formed by two transmembrane segments (TM1 and TM2) with its C and N terminal, located intracellularly and joined by a large extracellular loop. In the CNS, ASIC1a, ASIC2a, and ASIC2b, which are arranged in homo- and hetero-trimeric complexes, form most ASIC channels. The ASICs have been found to be evenly distributed in neurons, although concentrated in synaptic regions and anchored to postsynaptic density scaffolding proteins (Zha et al., 2009). The very fast gating kinetics of the ASICs and the Ca^2+^ blocking action led to the proposal that the ASIC3 channel opening was triggered because H^+^, displacing Ca^2+^, relieves the blockage of the channel (Immke and McCleskey, 2003). Recent evidence shows that a ring of rat ASIC3 glutamates, located above the channel gate, modulates proton sensitivity and contributes to the Ca^2+^ block site. Mutations of this site reduce Ca^2+^ block of the channel, making it similar to ASIC1a (Zuo et al., 2018). In chicken ASIC1a, the most thoroughly studied of the ASICs, the gating of the channel induces a displacement of the TM2 segment, opening the pore like a diaphragm and allowing ions to pass through a selectivity filter formed by G-A-S motifs from each of three adjacent subunits (Baconguis et al., 2014). The selectivity for monovalent cations of the filter is Li^+^ ≈ Na^+^>K^+^Rb^+^>Cs^+^ (Yang and Palmer, 2014). The permeability of the ASIC1a to Ca^2+^ has been found to be higher than other ASICs which are nearly impermeable to Ca^2+^ (Yermolaieva et al., 2004); but reports of the permeability ratio PNa/PCa for ASIC1a are conflicting, since a large variability ranging from 2.5 to 18.5 has been found (Bässler et al., 2001; Chu et al., 2002; Zhang and Canessa, 2002; Canessa, 2015). In cells transfected with ASIC1a, as well as heteromeric ASIC1a and ASIC2a and ASIC2b, it was found that PNa/PCa for ASIC1a was 1.8, for ASIC2a/1a it was 25.5 and for ASIC2b/1a it was 4.1 (Sherwood et al., 2011).

There is still a question about how cations reach the extracellular vestibule of the ASIC channel (Yang et al., 2018). The channel pore profile is formed by three interconnected vestibules forming a pathway for cations to reach the extracellular vestibule and cross the membrane when the channel opens (**Figure 2**; Yoder et al., 2018). The extracellular vestibule has three large fenestrations from which cations most likely enter (Gründer and Chen, 2010). Gating of the channel produces an expansion of the extracellular vestibule and reduction of lateral fenestrations (Yoder et al., 2018). Molecules interacting with the fenestration will act as partial blockers of the current, and molecules interacting with the central pore may produce a similar effect. Alas, the definition of the full ion permeability path, or of the relative contribution of the lateral fenestration, or the central pore, seems relevant to determine the action mechanism of molecules binding in the ASICs.

One significant question that has been put forward is whether or not the protons are the only and sufficient endogenous ligand for ASIC activation. The idea that proton activation of ASICs is a byproduct, and that a real endogenous activator is a “large neurotransmitter like” substance has been tested (Yu et al., 2010). However, until now, protons remain as the sole and most potent endogenous direct agonist of ASICs. The recent definition of the gating mechanism of chicken ASIC1a supports the idea that protons interacting with the acid pocket are the agonists for ASIC activation (Jasti et al., 2007; Gonzales et al., 2009; Vullo et al., 2017; Yoder et al., 2018). However, some elements out of the physiological range of ASIC2a pH_50_ activation of about 4.5–4.9 (Hesselager et al., 2004) suggest that something else will activate this channel. There are various endogenous modulators and partial agonists of the ASICs, but none known to physiologically activate the ASICs (Ortega-Ramírez et al., 2017). The 2-guanidine-4-methylquinazoline (GMQ) modulates ASIC3 at pH < 7.4 through a binding site distinct from the proton sensor (Yu et al., 2010). The MitTx, which is a viper toxin, evolved as a cytotoxin that, by maintaining the ASIC1a and 1b in the open state, produces cell damage (Bohlen et al., 2011). MitTx also modulates ASIC2a pH sensitivity. Recently, lindoldhamine, an alkaloid from *Laurus nobilis*, was shown to activate the ASIC3 in a proton-independent form and to act as a positive allosteric modulator of human and rat ASIC3 channels (Osmakov et al., 2018).

## Membrane Sensors for pH. Other Than ASICs

Extracellular pH changes modulate diverse cellular processes such as neuronal excitability, neurotransmitter release and postsynaptic responses, because pHe modulates the activity of different neuronal ion channels apart from the specific H^+^ sensing channels, including voltage-dependent Ca^2+^, K^+^, and Na^+^ channels, glutamate and GABA_R_, and Transient Receptor Potential-1 (TRPV1), among others (Chesler, 2003; Chiacchiaretta et al., 2017). The questions that arise are how pH influences channel activity, and what the physiological relevance of this channel modulation is. The slight acidification of the synaptic cleft modulates voltage-gated Ca^2+^ and Na^+^ channels in two ways, first, protons alter charged amino acids near the pore, thus reducing channel conductance. The H^+^ shifts the voltage dependence to more positive potentials, and in the case of Na^+^ channels, an alkaline medium lightly enhances the current (Tombaugh and Somjen, 1996). The sensibility of Ca^2+^ channels to pHe constitutes a significant element of proton signaling in the CNS, implicated both in vision and auditory function as described in the following section.

The two-pore domain K^+^ channels are essential for stabilizing the resting potential in most neurons, their activation produces a time- and voltage-independent K^+^ background current. These channels are usually inhibited by acidosis TASK 1 (two-pore domain K^+^ channel 1), TASK3, TASK2, TWIK (tandem of P-domain in a weak inwardly rectifying K ^+^ cannel) and TREK1 (TWIK-related K^+^ channel 1) and potentiated by extracellular alkalosis, except for TREK2 channels that are activated by a small pH drop (within 7.2 to 7.4) (Ehling et al., 2015). The physiological importance of pHe regulation of these channels continues to increase, it has been shown that under acid pHe conditions, the TASK and TWIK channels can even change their ionic selectivity and become permeable to Na^+^ (Ma L. et al., 2012). Additionally, the family of inward rectifier K^+^ channels (Kir) contributes to the leak K^+^ conductance in neurons and Kir conductance decreases with the acidification of the extracellular mediums, contributing to neuron depolarization (Coetzee et al., 1999).

**FIGURE 3:**
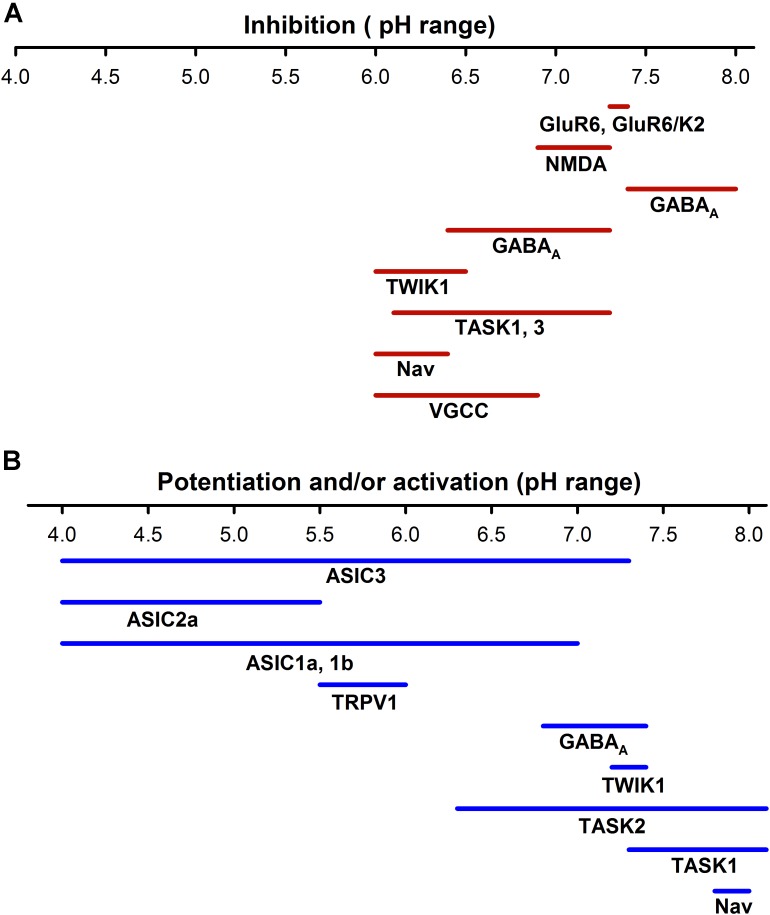
Sensitivity to pH of different neuronal ion channels. **(A)** Inhibition by pH of different neural ionic channels span from a pH of about 6.0 to 8.0, which indicates that at normal pH (7.4) a certain percentage of receptors such as KAR, AMPAR, or NMDAR are partially blocked. **(B)** ASICs are activated from a range of pHs from <7.8 to 4.0; in contrast the TRPV1 channels are much less sensitive to pH. Other channels such as voltage -gated K^+^, Na^+^, and Ca^2+^ channels can increase their activity at more alkaline pHs. The potentiation or activation of different channels has a much higher range (pH 4–8.0) than inhibition.

Ionotropic neurotransmitter receptors, including GABA(A) receptor and NMDA_R,_ α-amino-3-hydroxy-5-methyl-4-isoxazole propionic acid receptors (AMPA_R_), and Kainate receptors (KA_R_), are also modulated by pHe. The glutamate receptors are involved in neuronal development, synaptic plasticity, memory formation, and excitatory synaptic transmission (Traynelis et al., 2010). It has been reported that small drops in the pHe (pH 6.9–7.3) could reduce NMDA_R_ activity, except for recombinant NMDA N1/N3A that is strongly enhanced by acidification (Traynelis and Cull-Candy, 1990; Cummings and Popescu, 2016; **Figure 3**). Mutagenesis analysis indicates that critical residues for gaiting in these receptors regulate the pHe sensitivity of NMDA_R_, reducing their open probability in acid pHe (Low et al., 2003; Dravid et al., 2007). The effect of pHe on KA_R_ is voltage-independent and subunit dependent. The KA_R_ consists of 5 subunits (GluR5, GluR6, GluR5, K1, and K2) that combine in homo or heteromeric channels. Almost all kainate receptors are inhibited by protons, with the exception of the heteromeric GluR6/KA1 receptor, which is expressed in presynaptic neurons and potentiated by acid pH. At pH 7.3–7.4, homomeric GluR6 and heteromeric GluR6/KA2 are inhibited at Δ 20–25%, while GluR6/KA1 is enhanced to around 30% (Mott et al., 2003). In contrast, AMPA_R_ are much less extracellular proton sensitive (half-maximal inhibition at 6.1) and inhibition is due to enhanced desensitization of the AMPA_R_ (Ihle and Patneau, 2000).

The activation of ionotrpic GABA(A) receptor produce an inward Cl^-^ current. However, GABA(A) also seems to conduce bicarbonate from intra to extracellular space that leads to hyperpolarization of the postsynaptic neurons and alkalization of synaptic cleft (Ma B.F. et al., 2012). Reported regulation of GABA(A) by pHe at the postsynaptic level is highly variable. In hypothalamic neurons, acid pHe (∼6.4) inhibits GABA(A) current elicited by 10 μM GABA to 66.7 ± 6.8 %, whereas in alkaline pHe (8.4) increases to 212 ± 32.5% (Chen and Huang, 2014). In contrast, GABA(A) receptors in cultured cerebellar granule cells reduce their activity in alkaline pHe and are enhanced in acid pHe (Dietrich and Morad, 2010). The variability in the response of this receptor to pHe changes may be accounted for by the extremely variable experimental conditions in which they have been studied (experiments been carried out in native and recombinant GABA(A) at different concentrations of GABA and the presence or absence of buffers, such as HEPES).

Extracellular concentration of H^+^ may also activate some channels like the TREK2 K^+^ channels described above, and the TRPV1. The TRPV1 are non-selective cation channels activated by voltage, heat (>43°C), low pH (<6) and by several endogenous ligands (capsaicin, anandamide, and other endovanilloids). They are expressed throughout the CNS mainly in cortex, hippocampus, dentate gyrus, hypothalamus and superior colliculus (Toth et al., 2005). It has been suggested that they contribute to complex brain functions such as addiction, cognition and mood (Gibson et al., 2008; Tian et al., 2010, 2018; You et al., 2012). TRPV1 also localizes to synapses, and it has been proposed that it can modulate neurotransmission, synaptic plasticity and neuronal survival (Ho et al., 2012; Martins et al., 2014). In the dentate gyrus and nucleus accumbens, postsynaptic activation of TRPV1 from anandamide causes long term depression (Chavez et al., 2010; Grueter et al., 2010). TRPV1 are also implicated in neurodegeneration, in mesencephalic neuronal cultures and cortical microglia, and over-activation of TRPV1 raises intracellular Ca^2+^, producing mitochondrial damage and apoptosis (Kim et al., 2006).

The various proton sensitive channels, along with proton transporter activity, exchangers and buffer capacity always constitute a confusion variable in experiments that analyze the role of ASICs in the proton signaling mechanism in the nervous system. Their potential role and activation and inhibition by protons should always be considered an alternative explanation and a variable that should be controlled to ascertain the ASIC role in pHe actions.

## Evidence of the Role of H^+^ in Synaptic Transmission

Since the discovery of pHe sensitive responses in neurons, it was speculated that a proton concentration rise in extracellular mediums may activate a specific signaling system (Krishtal et al., 1987). The first solid evidence indicating the role of protons as an intercellular synaptic messenger was derived from experiments in *C. Elegans* in which it was shown that a H^+^ concentration rise by PBO-4 (a putative Na^+^/H^+^ ion exchanger) expressed in the lateral membrane of the intestine is enough to induce intestinal muscle contraction (Beg et al., 2008). Oscillatory transepithelial proton concentration regulates rhythmic behavior of the defecator program of *C. Elegans* (Pfeiffer et al., 2008). Further evidence showing the role of protons in synaptic transmission was obtained in the vertebrate retina, where it was shown that protons are the elusive mediator of lateral inhibition between horizontal cells and photoreceptors. Proposed mechanisms of lateral inhibition include GABA, protons, or an ephaptic mechanism (Kramer and Davenport, 2015). However, protons acting on Ca^2+^ channels have gained support as the mechanism that account for lateral inhibition, in fact, the use of genetically encoded pH sensors showed that L-type Ca^2+^ channels at the synaptic cleft are the sensors for protons (Wang et al., 2014). In the cone to horizontal neurons, synaptic cleft acidification implies the Na^+^-H^+^ exchangers as the main source of protons, and activity of HCO_3_^-^ transport in the horizontal cells will produce alkalization during light-evoked photoreceptor hyperpolarization (Warren et al., 2016). Notably in this case, similar to that of *C. elegans*, acidification is most probably mediated by the activity of an exchanger mechanism, specifically an NHE whose identity is not yet defined. It is worth noting that horizontal cells in the retina use GABA as a neurotransmitter, therefore in this case there is a segregation of GABA release and protons, both functioning in the same synapse.

Regarding the ASIC expression in the retina, the ASIC1a was found in cone photoreceptors, horizontal cells, some amacrine, and bipolar cells, and in the ganglion cell layer. Knockdown of ASIC1a or its blockade by PcTx1 decreased the photopic a- and b-waves and oscillatory potentials of the electroretinogram (Ettaiche et al., 2006). The ASIC3 is also expressed in the rod inner segment of photoreceptors, in horizontal cells, and some amacrine cells and in ganglion neurons. In early life (2–3 months) ASIC3 knockout mice show an increase in scotopic electroretinogram, but older mates (8 months) show a significant reduction in electroretinogram a- and b-waves, and disorganization of retina with degenerations of rod inner segments (Ettaiche et al., 2009).

In the auditory and vestibular system, in mice, the cochlear spiral ganglion neurons (SGNs) elicit a proton-gated ionic current that may be relevant in the response to high intensity auditory stimuli, since knockout of ASIC2 (including the ASIC2a and ASIC2b) exhibits increased resistance to noise-induced temporary threshold shifts, indicating a function of ASIC2 in hearing and the potentially harmful effects of acidosis (Peng et al., 2004). The SGNs and the organ of Corti of mice express ASIC3, and knockout of ASIC3 developed early hearing loss at about four months of age (Hildebrand et al., 2004). The ASIC1b subunit was detected in SGNs and at the insertion point of the stereocilia into the cuticular plate in the outer hair cells of the cochlea (Ugawa et al., 2006). In the rat vestibule, ASIC1b, and 4 were cloned and cDNA amplified (Gründer et al., 2000; Bässler et al., 2001). The ASICs have been shown to be expressed both in the rodent vestibular and cochlear afferent neurons (Mercado et al., 2006; González-Garrido et al., 2015). Expression of ASIC1a and ASIC2a was found in small vestibular ganglion neurons and afferent fibers in the utricle and crista stroma of the rat. The ASIC2b, ASIC3, and ASIC4 were expressed to a lesser extent (Mercado et al., 2006). The discharge of the vestibular system primary afferent neurons is highly sensitive to external pH changes and ASIC antagonists, such as amiloride and acetylsalicylic acid. These factors significantly reduced the vestibular-nerve discharge, corroborating that ASICs participate in the establishment of the afferent-resting discharge (Mercado et al., 2006; Vega et al., 2009). FMRF-amide was demonstrated to be present in calix ending synapses in the vestibular neuroepithelia, and FMRFamide perfusion increased the activity of the afferent neurons of the semicircular canal, indicating that ASIC currents are tonically active in resting condition (Mercado et al., 2012). In fact, it has been demonstrated that low pH perfusion may be enough to activate the action potential discharge in vestibular and cochlear afferent neurons. Based on these results, it has been proposed that ASICs mediate a synaptic input to cochlear and vestibular afferent neurons (Soto et al., 2014). In adult zebra fish, ASIC1 and ASIC4 were also found to be expressed in the hair cells of neuromasts, and ASIC2 in the afferent neurons, indicating the potential role for these ion channels in mechanosensation and postransductional sensory processing of movement information (Abbate et al., 2016).

**FIGURE 4:**
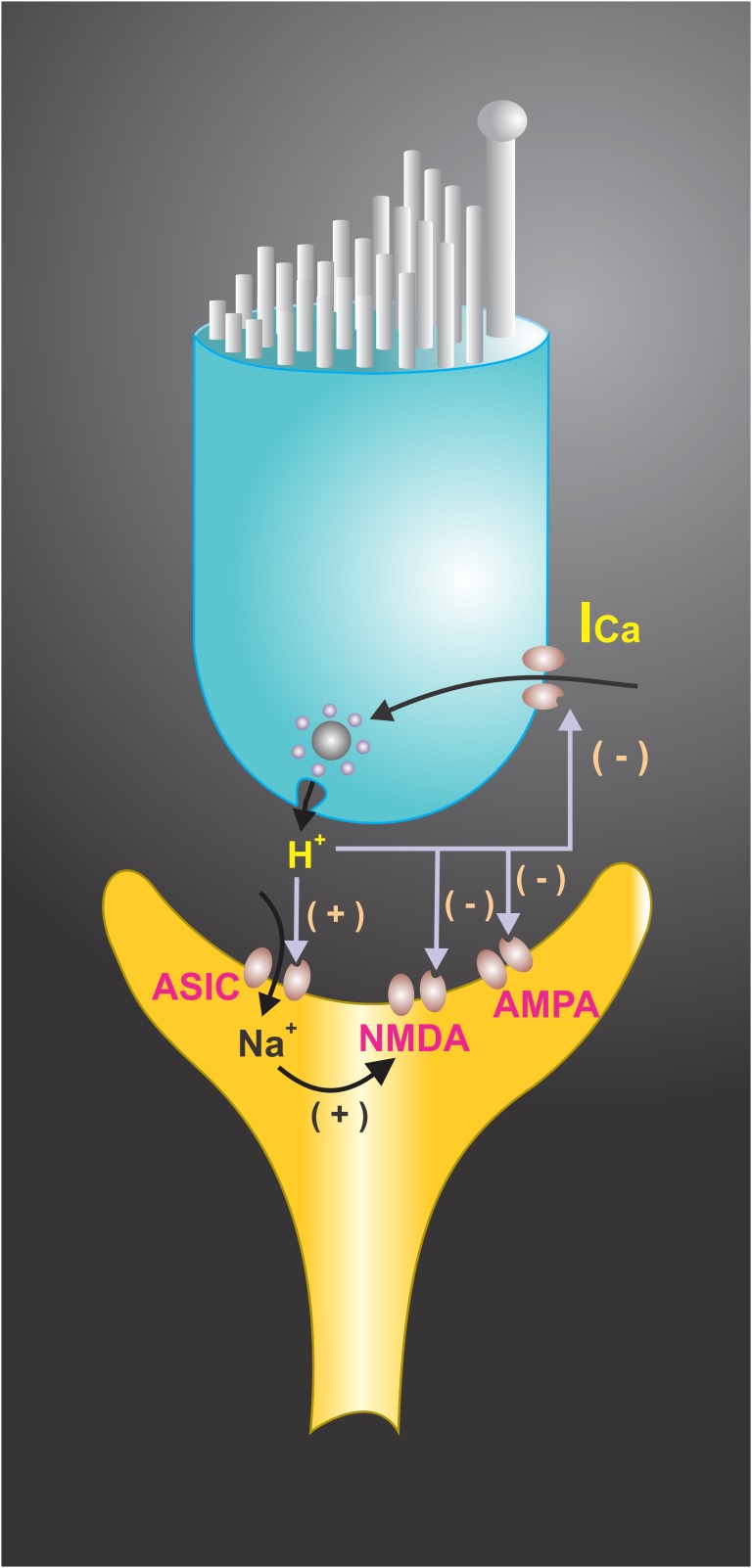
Extracellular protons have been shown to modulate voltage-activated ionic channels in hair cell receptoneural junctions. Presynaptic K^+^ and Ca^2+^ currents are modulated by H^+^, suggesting that they may function as a synaptic feedback mechanism in hair cells. A shift in the voltage dependence of the Ca^2+^ current to a more positive membrane potential was achieved at pH < 6.8. Extracellular pH also modulates the NMDA and AMPA receptors response to afferent transmitters and interacts with ASICs located at the synaptic endings, contributing to EPSC. The end result of H^+^ interactions with ionic channels may boost the postsynaptic response and restrict the release of neurotransmitters.

Paired recordings of afferent neurons and hair cells in bullfrog amphibian papillae showed that presynaptic Ca^2+^ current has a sag after the activation, which coincides with neurotransmitter release from hair cells. The sag in the current was shown to be produced by proton accumulation within the basolateral region of the cell where most of the Ca^2+^ channels are located, thus constituting a negative feedback system (Cho and von Gersdorff, 2014). This shows that a pH drop in synaptic endings can activate the ASICs which contribute to the EPSC, but at the same time contribute to presynaptic Ca^2+^ current decreases, limiting transmitter release. It has also been found that a drop of the pHe decreases K^+^ currents in isolated hair cells from the rat semicircular canals (Almanza et al., 2008). Therefore, depending on the balance, timing of the ASIC activation, and the decrease of the Ca^2+^ and the K^+^ currents, the acidification of synaptic cleft in the hair cell systems may boost the postsynaptic response and restrict the release time of neurotransmitters (**Figure 4**; Almanza et al., 2008). In the sensory neuroepithelium of the lagena obtained from the turtle, the activation of hair cells may induce pH changes in the basal side of neurosensory epithelia, and modeling data indicates that vesicle release may account for the pH drop in this microdomain (Highstein et al., 2014).

It is worth noting that the rapid deactivation kinetics and slow desensitization of the ASIC currents endow ASIC mediated responses with the capability of following high frequencies without any loss of response. Recombinant ASIC1a homomers and ASIC1a/2a heteromers, as well as native ASICs of DRG neurons, follow trains of brief pH 8.0 to 5.0 stimuli at high frequencies (> 50 Hz) without any loss of response amplitude or kinetic characteristics. Compared to glutamate evoked responses, they show a capacity for high-frequency signaling when other receptors desensitize (MacLean and Jayaraman, 2016). This makes ASICs ideal candidates for high frequency responses needed for sensory coding in cochlear afferent neurons (Fettiplace, 2017).

Interestingly, it has been shown that accumulation of protons due to hypoxia, and activation of anaerobic mechanisms in the inner ear may finally induce an activation of the vestibular afferent neurons, expressing ASICs and the induction of movement. This mechanism may be part of the processes which induce a person to move when there is a hypoxic condition. The role of this mechanism in Sudden Infant Death Syndrome (SIDS) has been considered as potentially relevant (Allen et al., 2013; Ramirez et al., 2016). Also, a high expression level of NHE in the brain stem is associated with an increase in incidence of deaths by SIDS (Wiemann et al., 2008). This last mechanism will be related to the pH modulation of the excitability of respiratory control neurons.

A significant role of H^+^ as co-transmitter signaling is found in the auditory pathway. The postsynaptic neurons of the medial nucleus of the trapezoid body (MNTB) at the mouse calyx of Held synapse express functional homomeric ASIC1a channels that can be activated by protons co-released from synaptic vesicles (González-Inchauspe et al., 2017). Currents evoked by acid pHe perfusion were blocked by PcTx1 and in ASIC1a^-/-^ mice. Most relevant is the fact that postsynaptic potentials produced by presynaptic stimulation are of a magnitude sufficient to evoke action potentials in postsynaptic neurons of the MNTB in absence of glutamate receptor activation. High frequency stimulation of presynaptic terminals leads to Ca^2+^ increase in MNTB neurons. The lack of functional ASICs during high frequency stimulation enhances short-term depression of glutamatergic EPSCs. These results demonstrate that presynaptic co-release of protons modulate synaptic transmission by activating ASIC1a at the calyx of Held-MNTB synapse (González-Inchauspe et al., 2017).

In other sensory systems, such as pain pathways, ASICs are widely expressed both in the peripheral and central nervous systems (Deval et al., 2010; Wemmie et al., 2013). ASIC3 is highly expressed in DRG neurons, mediating pain associated with a decrease of pHe in processes such as inflammation, ischemia and cancer. The ASIC3 also mediates the mechanical hyperalgesia associated with muscle inflammation (Sluka et al., 2003, 2007), although most of the DRG neurons (including cutaneous afferents) express ASICs that probably contribute to pain processing in various modalities (Ortega-Ramírez et al., 2017). In the brain, intracerebroventricular injections of PcTx1 attenuate acute pain responses, as well as pain behavior in chronic inflammatory and neuropathic pain models (Mazzuca et al., 2007), Also, mambalgin-1, an ASIC1a blocker, attenuates pain behavior due to the inhibition of heteromeric ASIC1a/2a in the spinal dorsal horn neurons in an opioid-independent form (Baron et al., 2008). Moreover, suppression of ASIC1a attenuates both mechanical and thermal hypersensitivity induced by peripheral inflammation. The role of ASICs in pain processing is further supported by the high level of expression of ASIC1a in multiple brain regions associated with pain, such as the ventral and dorsal regions of periaqueductal gray matter (Wemmie et al., 2003). These results show that ASICs are essential for pain system activation at spinal and supraspinal levels.

Expression and activation of ASICs in brain areas involved in motor behavior and sensitivity to various psychostimulants such as cocaine, morphine and amphetamines have suggested that ASICs play an important role in addictive behavior (Suman et al., 2010; Jiang et al., 2013). Chronic exposure to cocaine increases the expression of ASIC1 and ASIC2 in the striatum (both caudate putamen and nucleus accumbens -NAc-) (Zhang et al., 2009), and overexpression of ASIC1a in the NAc reduces the self-administration of cocaine in rats. The suppression of ASIC1a increases the conditioned place preference produced by cocaine and morphine (Kreple et al., 2014). At the synaptic level, EPSC in medium spinal neurons (MSN) in the NAc was increased when the buffer capacity in the extracellular medium was reduced, in contrast, the use of amiloride (an unspecific ASIC antagonist) reduces EPSC. ASIC1a knockout mice showed alterations in dendritic spine morphology and frequency of EPSC, suggesting that ASIC1a can regulate excitatory synaptic transmission in the NAc, supporting the hypothesis that H^+^ co-release with glutamate significantly contributes to synaptic input by activation of the ASICs and contributes to a decrease in addictive behavior (Kreple et al., 2014).

In the lateral amygdala, it was found that ASIC1 expression is significantly higher than in other areas of the CNS, and in ASIC1 knockout mice the H^+^ evoked currents of amygdala neurons and also fear conditioning were undetectable (Wemmie et al., 2003). The rise in the extracellular proton concentration, secondarily to sustained activation of neurons, is at the basis of fear response and fear conditioning in lateral amygdala pyramidal neurons, contributing to EPSC (Du et al., 2014). The neurons are initially activated by glutamatergic input, so, the effect is due to a positive feedback between pHe neuron activity and ASICs activation. Other pHe-related mechanisms, such as membrane pump expression and abundance of mobile buffer in the media, along with aerobic capability of the neurons, would have a significant role in the acidification of extracellular media. The reaction between CO_2_ and water catalyzed by carbonic anhydrase generates large H^+^ concentration changes, in fact, inhaling CO_2_ can trigger panic attacks (Coryell et al., 2006), most likely because of the activation of ASIC currents in amygdala. Buffering pH attenuated fear behavior, and directly reduced pH with amygdala microinjections, reproduced the effect of CO_2_ (Ziemann et al., 2009). Interestingly, the effect mediated by pHe changes secondary to carbonic acid has been introduced in the computerized human nervous system function emulation (HNSFE) technology, which uses CO_2_ sensors to emulate the response of ASICs allowed to produce fearful emotional responses and complex avoidant behavior of an android (Frenger, 2010, 2017).

In the case of a convulsive crisis, the overactivation of neurons increases the extracellular H^+^ concentration and the activation of ASIC1a and ASIC3, whose expression in GABAergic inhibitory interneurons is larger than that in excitatory neurons, contributing to ending seizures. The kainate-induced seizures were longer and more severe in ASIC1 knockout mice. Consistent with the proposal that ASICs participate in ending seizures, the loss of ASIC1a also reduced postictal depression (Ziemann et al., 2008). Although ASIC3 brain expression is considered low, it was found to be expressed in inhibitory GABAergic interneurons and glial cells. A block of ASIC3 by APETx2 in pilocarpine- or pentylenetetrazole (PTZ)-induced seizures shortened the latency and increased the incidence of seizures (Cao et al., 2014). Thus, the increase in extra pH leads to increased activity, mainly in inhibitory neurons, finally limiting the neuronal discharge by the release of inhibitory neurotransmitter. The ending of a convulsive crisis has been a mystery in clinical neurosciences. It was always thought that some metabolic mechanism was critical for ending crisis, but no exact mechanism was devised until the ASIC was discovered. However, an opposite effect of ASICs in epilepsy has also been found, ASIC2a overexpression resulted in increased hippocampal seizure susceptibility (Wu H. et al., 2016). In fact, amiloride delays the onset of pilocarpine-induced seizures in rats (N’Gouemo, 2008). Brain hypometabolism is a common finding in patients and in animal models of epilepsy, hippocampal glucose hypometabolism elevates ASIC2a expression by suppressing Transcription factor CP2 expression, which further enhances the excitability of CA1 pyramidal neurons and seizure susceptibility in patients with temporal lobe epilepsy (Zhang et al., 2017). Zhang et al. (2017) have proposed that before seizure onset, increased ASIC2a expression could increase neuronal excitability. As large quantities of lactic and glutamic acid are released during seizures, extracellular H^+^ accumulation activates ASIC1a and ASIC3, causing GABA release from interneurons and ending the seizure.

## Conclusion and Perspectives

In the near future, we will get information and develop a whole picture of networks of functional interactions among membrane proteins including receptors, transporters and ionic channels. A channelome picture of the cell at the micro- and nano-domains will allow us to understand channel function and its network and mutual modulation, coupled ionic fluxes, membrane potential and osmotic and diffusional forces, interacting all together to determine cell excitability and cell communication.

Proton signaling implies a paradigm shift in relation to neurotransmission and neuromodulation in the CNS. Metabolic activity and the hydrolysis of ATP produce a constitutive and non-regulated release of protons in the extracellular space, which constitute a form of volume neurotransmission and, as described above, may play a key role in neuron excitability regulation. Examples of H^+^ mediated signaling indicate a restricted co-transmitter role for protons at the synaptic level; there is also evidence of H^+^ and GABA transmitter segregation in horizontal neurons; and a modulatory role of H^+^ of metabolic origin also exists, but until now there is no evidence, in vertebrates, to show that there is a regulated Ca^2+^ dependent release of protons in synaptic endings; protons at synaptic endings are always co-released with some neurotransmitter. However, release and regulation of extracellular protons implies a complexity which seems to go farther than the standard neurotransmission and modulation concepts in the nervous system, constituting a new paradigm in cell signaling mechanisms.

## Author Contributions

ES conceived the article and its structure. ES, AO-R, and RV worked together to write the article.

## Conflict of Interest Statement

The authors declare that the research was conducted in the absence of any commercial or financial relationships that could be construed as a potential conflict of interest.

## Abbreviations

ΔμH^+^: Electrochemical gradient
ΔpH: H^+^ concentration gradient
Δψ: Electrical potential
AE: Anion Exchanger
AMPA_R_: α-amino-3-hydroxy-5-methyl-4-isoxazole propionic acid
ASD: Autism spectrum disorders
ASIC: Acid Sensing Ionic Channels
CAs: Carbonic anhydrase enzyme family
CHO: Chinese Hamster Ovary
CNS: Central nervous system
DEG: Degenerins
DRG: Dorsal root ganglion neurons
ENaC: Epithelial sodium channel
EPSC: Excitatory postsynaptic current
GABA: Gamma aminobutyric acid
GMQ: 2-guanidine-4-methylquinazoline
H^+^: Protons
HNSFE: Human nervous system function emulation
Hv1: Voltage-gated proton channel
KA_R_: Kainate receptors
Kir: Inward rectifier K^+^ channels
MCT: Monocarboxylate transporters
MNTB: Medial nucleus of the trapezoid body
MSN: Medium spinal neurons
NAc: Nucleus accumbens
NCBTs: Na^+^-coupled HCO3 transporters
NHE: Na^+^/H^+^ exchangers
NMDAR: N-methyl-D-aspartate receptors
pHe: Extracellular pH
pHi: Intracellular pH
PTZ: Pentylenetetrazole
SGNs: Spiral ganglion neurons
SIDS: Sudden infant death syndrome
SV: Synaptic vesicles
TASK1, TASK2: Two-pore domain K^+^ channel
TM1 and TM2: Transmembrane segments 1 and 2
TREK1: TWIK-related K^+^ channel 1
TRPV1: Transient Receptor Potential-1
TWIK: Tandem of P-domain in a weak inwardly rectifying K^+^ cannel
V-ATPase: Vacuolar H^+^-ATPase
VGAT: Vesicular GABA transporters
VGCC: Voltage gated calcium channels
VGLUT: Vesicular glutamate transporters

## References

[B1] Abbate F. Madrigrano M. Scopitteri T. Levanti M. Cobo J. L. Germanà A. (2016). Acid-sensing ion channel immunoreactivities in the cephalic neuromasts of adult zebrafish. *Ann. Anat.* 207 27–31. 10.1016/j.aanat.2016.06.007 27443821

[B2] Allen T. Garcia A. J. III Tang J. Ramirez J. M. Rubens D. D. (2013). Inner ear insult ablates the arousal response to hypoxia and hypercarbia. *Neuroscience* 253 283–291. 10.1016/j.neuroscience.2013.08.059 24021919 PMC3988803

[B3] Almanza A. Mercado F. Vega R. Soto E. (2008). Extracellular pH modulates the voltage-dependent Ca2^+^ current and low threshold K^+^ current in hair cells. *Neurochem. Res.* 33 1435–1441. 10.1007/s11064-007-9565-9 18259861

[B4] Alvadia C. M. Sommer T. Bjerregaard-Andersen K. Damkier H. H. Montrasio M. Aalkjaer C. (2017). The crystal structure of the regulatory domain of the human sodium-driven chloride/bicarbonate exchanger. *Sci. Rep.* 7:12131. 10.1038/s41598-017-12409-0 28935959 PMC5608694

[B5] Askwith C. C. Wemmie J. A. Price M. P. Rokhlina T. Welsh M. J. (2004). Acid-sensing ion channel 2 (ASIC2) modulates ASIC1 H^+^-activated currents in hippocampal neurons. *J. Biol. Chem.* 279 18296–18305. 10.1074/jbc.M312145200 14960591

[B6] Baconguis I. Bohlen C. J. Goehring A. Julius D. Gouaux E. (2014). X-ray structure of acid-sensing ion channel 1-snake toxin complex reveals open state of a Na^+^-selective channel. *Cell* 156 717–729. 10.1016/j.cell.2014 24507937 PMC4190031

[B7] Baron A. Voilley N. Lazdunski M. Lingueglia E. (2008). Acid sensing ion channels in dorsal spinal cord neurons. *J. Neurosci.* 28 1498–1508. 10.1523/JNEUROSCI.4975-07.2008 18256271 PMC6671562

[B8] Bässler E. L. Ngo-Anh T. J. Geisler H. S. Ruppersberg J. P. Grunder S. (2001). Molecular and functional characterization of acid-sensing ion channel (ASIC) 1b. *J. Biol. Chem.* 276 33782–33787. 10.1074/jbc.M104030200 11448963

[B9] Beg A. A. Ernstrom G. G. Nix P. Davis M. W. Jorgensen E. M. (2008). Protons act as a transmitter for muscle contraction in *C. elegans*. *Cell* 132 149–160. 10.1016/j.cell.2007.10.058 18191228 PMC2258244

[B10] Bohlen C. J. Chesler A. T. Sharif-naeini R. Medzihradszky K. F. Zhou S. King D. (2011). A heteromeric Texas coral snake toxin targets acid-sensing ion channels to produce pain. *Nature* 479 410–414. 10.1038/nature10607 22094702 PMC3226747

[B11] Boiko N. Kucher V. Wang B. Stockand J. D. (2014). Restrictive expression of acid-sensing ion channel 5 (Asic5) in unipolar brush cells of the vestibulocerebellum. *PLoS One* 9:e91326. 10.1371/journal.pone.0091326 24663811 PMC3963869

[B12] Brett C. L. Donowitz M. Rao R. (2005). Evolutionary origins of eukaryotic sodium/proton exchangers. *Am. J. Physiol. Cell Physiol.* 288 C223–C239. 10.1152/ajpcell.00360.2004 15643048

[B13] Canessa C. (2015). “Acid sensing ion channels,” in *Handbook of Ion Channels*, eds Zheng J. Trudeau M. C. (Boca Raton, FL: CRC Press), 383–394. 10.1201/b18027-29

[B14] Cao Q. Wang W. Gu J. Jiang G. Wang K. Xu Z. (2014). Elevated expression of acid-sensing ion channel 3 inhibits epilepsy via activation of interneurons. *Mol. Neurobiol.* 53 485–498. 10.1007/s12035-014-9014-0 25476599

[B15] Carafoli E. (2005). Calcium-a universal carrier of biological signals. Delivered on 3 July 2003 at the Special FEBS Meeting in Brussels. *FEBS J.* 272 1073–1089. 10.1111/j.1742-4658.2005.04546.x 15720383

[B16] Casey J. R. Grinstein S. Orlowski J. (2010). Sensors and regulators of intracellular pH. *Nat. Rev. Mol. Cell Biol.* 11 50–61. 10.1038/nrm2820 19997129

[B17] Chavez A. E. Chiu C. Q. Castillo P. E. (2010). TRPV1 activation by endogenous anandamide triggers postsynaptic long-term depression in dentate gyrus. *Nat. Neurosci.* 13 1511–1518. 10.1038/nn.2684 21076423 PMC3058928

[B18] Chen X. Kalbacher H. Gründer S. (2006). Interaction of acid-sensing ion channel (ASIC) 1 with the tarantula toxin psalmotoxin 1 is state dependent. *J. Gen. Physiol.* 127 267–276. 10.1085/jgp.200509409 16505147 PMC2151504

[B19] Chen X. Whissell P. Orser B. A. MacDonald J. F. (2011). Functional modifications of acid-sensing ion channels by ligand gated chloride channels. *PLoS One* 6:e21970. 10.1371/journal.pone.0021970 21789198 PMC3138761

[B20] Chen Z. L. Huang R. Q. (2014). Extracellular pH modulates GABAergic neurotransmission in rat hypothalamus. *Neuroscience* 271 64–76. 10.1016/j.neuroscience.2014.04.028 24780768 PMC4067938

[B21] Chesler M. (2003). Regulation and modulation of pH in the brain. *Physiol. Rev.* 83 1183–1221. 10.1152/physrev.00010.2003 14506304

[B22] Chiacchiaretta M. Latifi S. Bramini M. Fadda M. Fassio A. Benfenati F. (2017). Neuronal hyperactivity causes Na^+^/H^+^ exchanger-induced extracellular acidification at active synapses. *J. Cell Sci.* 130 1435–1449. 10.1242/jcs.198564 28254883

[B23] Cho S. von Gersdorff H. (2014). Proton-mediated block of Ca2^+^ channels during multivesicular release regulates short-term plasticity at an auditory hair cell synapse. *J. Neurosci.* 34 15877–15887. 10.1523/JNEUROSCI.2304-14.2014 25429130 PMC4244462

[B24] Chu X. P. Miesch J. Johnson M. Root L. Zhu X. M. Chen D. (2002). Proton-gated channels in PC12 cells. *J. Neurophysiol.* 87 2555–2561. 10.1152/jn.00741.2001 11976391

[B25] Coetzee W. A. Amarillo Y. Chiu J. Chow A. Lau D. McCormack T. (1999). Molecular diversity of K^+^ channels. *Ann. N. Y. Acad. Sci.* 868 233–285. 10.1111/j.1749-6632.1999.tb11293.x 10414301

[B26] Coryell W. Pine D. Fyer A. Klein D. (2006). Anxiety responses to CO2 inhalation in subjects at high-risk for panic disorder. *J. Affect. Disord.* 92 63–70. 10.1016/j.jad.2005.12.045 16527360

[B27] Cristofori-Armstrong B. Rash L. D. (2017). Acid-sensing ion channel (ASIC) structure and function: insights from spider, snake and sea anemone venoms. *Neuropharmacology* 127 173–184. 10.1016/j.neuropharm.2017.04.042 28457973

[B28] Cummings K. A. Popescu G. K. (2016). Protons potentiate GluN1/GluN3A currents by attenuating their desensitization. *Sci. Rep.* 6:23344. 10.1038/srep23344 27000430 PMC4802338

[B29] Daniel H. Spanier B. Kottra G. Weitz D. (2006). From bacteria to man: archaic proton-dependent peptide transporters at work. *Physiology* 21 93–102. 10.1152/physiol.00054.2005 16565475

[B30] Del-Bel E. De-Miguel F. F. (2018). Extrasynaptic neurotransmission mediated by exocytosis and diffusive release of transmitter substances. *Front. Synaptic Neurosci.* 10:13. 10.3389/fnsyn.2018.00013 29937726 PMC6003215

[B31] Deval E. Gasull X. Noël J. Salinas M. Baron A. Diochot S. (2010). Acid-sensing ion channels (ASICs): pharmacology and implication in pain. *Pharmacol. Ther.* 128 549–558. 10.1016/j.pharmthera.2010.08.006 20807551

[B32] Di Giulio M. (2005). Structuring of the genetic code took place at acidic pH. *J. Theor. Biol.* 237 219–226. 10.1016/j.jtbi.2005.04.009 15978625

[B33] Diering G. H. Numata M. (2014). Endosomal pH in neuronal signaling and synaptic transmission: role of Na^+^/H^+^ exchanger NHE5. *Front. Physiol.* 4:412 10.3389/fphys.2013.00412 PMC3888932 24454292

[B34] Diering G. H. Mills F. Bamji S. X. Numata M. (2011). Regulation of dendritic spine growth through activity-dependent recruitment of the brain-enriched Na^+^/H^+^ exchanger NHE5. *Mol. Biol. Cell* 22 2246–2257. 10.1091/mbc.e11-01-0066 21551074 PMC3128527

[B35] Dietrich C. J. Morad M. (2010). Synaptic acidification enhances GABAA signaling. *J. Neurosci.* 30 16044–16052. 10.1523/JNEUROSCI.6364-09.2010 21106843 PMC3073570

[B36] Donier E. Rugiero F. Jacob C. Wood J. N. (2008). Regulation of ASIC activity by ASIC4–new insights into ASIC channel function revealed by a yeast two-hybrid assay. *Eur. J. Neurosci.* 28 74–86. 10.1111/j.1460-9568.2008.06282.x 18662336

[B37] Donowitz M. Ming Tse C. Fuster D. (2013). SLC9/NHE gene family, a plasma membrane and organellar family of Na^+^/H^+^ exchangers. *Mol. Aspects Med.* 34 236–251. 10.1016/j.mam.2012.05.001 23506868 PMC3724465

[B38] Dravid S. M. Erreger K. Yuan H. Nicholson K. Le P. Lyuboslavsky P. (2007). Subunit-specific mechanisms and proton sensitivity of NMDA receptor channel block. *J. Physiol.* 581(Pt 1), 107–128. 10.1113/jphysiol.2006.124958 17303642 PMC2075223

[B39] Du J. Hossain Z. Mandal J. (2017). Protons: a neurotransmitter in the brain. *Edorium J. Cell Biol.* 3 1–3. 10.5348/C06-2017-5-ED-1

[B40] Du J. Reznikov L. R. Price M. P. Zha X. M. Lu Y. Moninger T. O. (2014). Protons are a neurotransmitter that regulates synaptic plasticity in the lateral amygdala. *Proc. Natl. Acad. Sci. U.S.A.* 111 8961–8966. 10.1073/pnas.1407018111 24889629 PMC4066526

[B41] Egashira Y. Takase M. Watanabe S. Ishida J. Fukamizu A. Kaneko R. (2016). Unique pH dynamics in GABAergic synaptic vesicles illuminates the mechanism and kinetics of GABA loading. *Proc. Natl. Acad. Sci. U.S.A.* 113 10702–10707. 10.1073/pnas.1604527113 27601664 PMC5035854

[B42] Ehling P. Cerina M. Budde T. Meuth S. G. Bittner S. (2015). The CNS under pathophysiologic attack—examining the role of K2P channels. *Pflügers Arch.* 467 959–972. 10.1007/s00424-014-1664-2 25482672

[B43] Eriksen J. Chang R. McGregor M. Silm K. Suzuki T. Edwards R. H. (2016). Protons regulate vesicular glutamate transporters through an allosteric mechanism. *Neuron* 90 768–780. 10.1016/j.neuron.2016.03.026 27133463 PMC4886649

[B44] Ettaiche M. Deval E. Cougnon M. Lazdunski M. Voilley N. (2006). Silencing acid-sensing ion channel 1a alters cone-mediated retinal function. *J. Neurosci.* 26 5800–5809. 10.1523/JNEUROSCI.0344-06.2006 16723538 PMC6675265

[B45] Ettaiche M. Deval E. Pagnotta S. Lazdunski M. Lingueglia E. (2009). Acid-sensing ion channel 3 in retinal function and survival. *Invest. Ophthalmol. Vis. Sci.* 50 2417–2426. 10.1167/iovs.08-3028 19117938

[B46] Farsi Z. Preobraschenski J. van den Bogaart G. Riedel D. Jahn R. Woehler A. (2016). Single-vesicle imaging reveals different transport mechanisms between glutamatergic and GABAergic vesicles. *Science* 351 981–984. 10.1126/science.aad8142 26912364

[B47] Fettiplace R. (2017). Hair cell transduction, tuning, and synaptic transmission in the mammalian cochlea. *Compr. Physiol.* 7 1197–1227. 10.1002/cphy.c160049 28915323 PMC5658794

[B48] Frenger P. (2010). Emulating a carbon dioxide trigger for the fear response. *Can. Med. Biol. Eng. Soc. CMEBS Proc.* 33 527–529.

[B49] Frenger P. (2017). Thirty-five years of artificial emotions: an extended case history. *Can. Med. Biol. Eng. Soc. CMEBS Proc.* 40 572–577.

[B50] Fuxe K. Dahlström A. Höistad M. Marcellino D. Jansson A. Rivera A. (2007). From the Golgi-Cajal mapping to the transmitter-based characterization of the neuronal networks leading to two modes of brain communication: wiring and volume transmission. *Brain Res. Rev.* 55 17–54. 10.1016/j.brainresrev.2007.02.009 17433836

[B51] Gibson H. E. Edwards J. G. Page R. S. Van Hook M. J. Kauer J. A. (2008). TRPV1 channels mediate long-term depression at synapses on hippocampal interneurons. *Neuron* 57 746–759. 10.1016/j.neuron.2007.12.027 18341994 PMC2698707

[B52] Gonzales E. B. Kawate T. Gouaux E. (2009). Pore architecture and ion sites in acid sensing ion channels and P2X receptors. *Nature* 460 599–604. 10.1038/nature08218 19641589 PMC2845979

[B53] González-Garrido A. Vega R. Mercado F. López I. A. Soto E. (2015). Acid-sensing ion channels expression, identity and role in the excitability of the cochlear afferent neurons. *Front. Cell. Neurosci.* 9:483. 10.3389/fncel.2015.00483 26733809 PMC4686812

[B54] González-Inchauspe C. Urbano F. J. Di Guilmi M. N. Uchitel O. D. (2017). Acid sensing ion channels activated by evoked released protons modulate synaptic transmission at the mouse calyx of Held synapse. *J. Neurosci.* 37 2589–2599. 10.1523/JNEUROSCI.2566-16.2017 28159907 PMC6596635

[B55] Grueter B. A. Brasnjo G. Malenka R. C. (2010). Postsynaptic TRPV1 triggers cell type-specific longterm depression in the nucleus accumbens. *Nat. Neurosci.* 13 1519–1525. 10.1038/nn.2685 21076424 PMC3092590

[B56] Gründer S. Chen X. (2010). Structure, function, and pharmacology of acid-sensing ion channels (ASICs): focus on ASIC1a. *Int. J. Physiol. Pathophysiol. Pharmacol.* 2 73–94. 21383888 PMC3047259

[B57] Gründer S. Geissler H. S. Bassler E. L. Ruppersberg J. P. (2000). A new member of acid-sensing ion channels from pituitary gland. *Neuroreport* 11 1607–1611. 10.1097/00001756-200006050-00003 10852210

[B58] Gründer S. Pusch M. (2015). Biophysical properties of acid-sensing ion channels (ASICs). *Neuropharmacology* 94 9–18. 10.1016/j.neuropharm.2014.12.016 25585135

[B59] Hanukoglu I. (2017). ASIC and ENaC type sodium channels: conformational states and the structures of the ion selectivity filters. *FEBS J.* 284 525–545. 10.1111/febs.13840 27580245

[B60] Hesselager M. Timmermann D. B. Ahring P. K. (2004). pH-dependency and desensitization kinetics of heterologously expressed combinations of ASIC subunits. *J. Biol. Chem.* 279 11006–11015. 10.1074/jbc.M313507200 14701823

[B61] Highstein S. M. Holstein G. R. Mann M. A. Rabbitt R. D. (2014). Evidence that protons act as neurotransmitters at vestibular hair cell–calyx afferent synapses. *Proc. Natl. Acad. Sci. U.S.A.* 111 5421–5426. 10.1073/pnas.1319561111 24706862 PMC3986198

[B62] Hildebrand M. S. de Silva M. G. Klockars T. Rose E. Price M. Smith R. J. (2004). Characterization of DRASIC in the mouse inner ear. *Hear. Res.* 190 149–160. 10.1016/S0378-5955(04)00015-2 15051137

[B63] Ho K. W. Ward N. J. Calkins D. J. (2012). TRPV1: a stress response protein in the central nervous system. *Am. J. Neurodegener. Dis.* 1 1–14. 22737633 PMC3560445

[B64] Ihle E. C. Patneau D. K. (2000). Modulation of α-amino-3-hydroxy-5-methyl-4-isoxazolepropionic acid receptor desensitization by extracellular protons. *Mol. Pharmacol.* 58 1204–1212. 10.1124/mol.58.6.1204 11093755

[B65] Immke D. C. McCleskey E. W. (2003). Protons open Acid-sensing ion channels by catalyzing relief of Ca2^+^ blockade. *Neuron* 37 75–84. 10.1016/S0896-6273(02)01130-3 12526774

[B66] Jasti J. Furukawa H. Gonzales E. B. Gouaux E. (2007). Structure of acid sensing ion channel 1 at 1.9 A resolution and low pH. *Nature* 449 316–323. 10.1038/nature06163 17882215

[B67] Jiang Q. Wang C. M. Fibuch E. E. Wang J. Q. Chu X. P. (2013). Differential regulation of locomotor activity to acute and chronic cocaine administration by acid-sensing ion channel 1a and 2 in adult mice. *Neuroscience* 246 170–178. 10.1016/j.neuroscience.2013.04.059 23644053 PMC3855427

[B68] Kellenberger S. Schild L. (2002). Epithelial sodium channel/degenerin family of ion channels: a variety of functions for a shared structure. *Physiol. Rev.* 82 735–767. 10.1152/physrev.00007.2002 12087134

[B69] Kim S. R. Kim S. U. Oh U. Jin B. K. (2006). Transient receptor potential vanilloid subtype 1 mediates microglial cell death in vivo and in vitro via Ca2^+^-mediated mitochondrial damage and cytochrome c release. *J. Immunol.* 177 4322–4329. 10.4049/jimmunol.177.7.4322 16982866

[B70] Kramer R. H. Davenport C. M. (2015). Lateral inhibition in the vertebrate retina: the case of the missing neurotransmitter. *PLoS Biol.* 13:e1002322. 10.1371/journal.pbio.1002322 26656622 PMC4675548

[B71] Kreple C. J. Lu Y. Taugher R. J. Schwager-Gutman A. L. Du J. Stump M. (2014). Acid-sensing ion channels contribute to synaptic transmission and inhibit cocaine-evoked plasticity. *Nat. Neurosci.* 17 1083–1091. 10.1038/nn.3750 24952644 PMC4115047

[B72] Krishtal O. A. Osipchuk Y. V. Shelest T. N. Smirnoff S. V. (1987). Rapid extracellular pH transients related to synaptic transmission in rat hippocampal slices. *Brain Res.* 436 352–356. 10.1016/0006-8993(87)91678-7 2829992

[B73] Krishtal O. A. Pidoplichko V. I. (1980). A receptor for protons in the nerve cell membrane. *Neuroscience* 5 2325–2327. 10.1016/0306-4522(80)90149-9 6970348

[B74] Krishtal O. A. Pidoplichko V. I. (1981). A “receptor” for protons in small neurons of trigeminal ganglia: possible role in nociception. *Neurosci. Lett.* 24 243–246. 10.1016/0304-3940(81)90164-6 6269026

[B75] Kusama N. Gautam M. Harding A. M. Snyder P. M. Benson C. J. (2013). Acid-sensing ion channels (ASICs) are differentially modulated by anions dependent on their subunit composition. *Am. J. Physiol. Cell Physiol.* 304 C89–C101. 10.1152/ajpcell.00216.2012 23135698 PMC3543573

[B76] Kusama N. Harding A. M. Benson C. J. (2010). Extracellular chloride modulates the desensitization kinetics of acid-sensing ion channel 1a (ASIC1a). *J. Biol. Chem.* 285 17425–17431. 10.1074/jbc.M109.091561 20385551 PMC2878506

[B77] Lefèvre C. M. Diakov A. Haerteis S. Korbmacher C. Gründer S. Wiemuth D. (2014). Pharmacological and electrophysiological characterization of the human bile acid-sensitive ion channel (hBASIC). *Pflugers Arch.* 466 253–263. 10.1007/s00424-013-1310-4 23842738

[B78] Li T. Yang Y. Canessa C. M. (2010). Leu85 in the beta1-beta2 linker of ASIC1 slows activation and decreases the apparent proton affinity by stabilizing a closed conformation. *J. Biol. Chem.* 285 22706–22712. 10.1074/jbc.M110.134114 20479002 PMC2903392

[B79] Li W. G. Xu T. L. (2011). ASIC3 channels in multimodal sensory perception. *ACS Chem. Neurosci.* 2 26–37. 10.1021/cn100094b 22778854 PMC3369706

[B80] Li X. Wu F. R. Xu R. S. Hu W. Jiang D. L. Ji C. (2014). Acid-sensing ion channel 1a-mediated calcium influx regulates apoptosis of endplate chondrocytes in intervertebral discs. *Expert Opin. Ther. Targets* 18 1–14. 10.1517/14728222.2014.859248 24261866

[B81] Lin S. H. Cheng Y. R. Banks R. W. Min M. Y. Bewick G. S. Chen C. C. (2016). Evidence for the involvement of ASIC3 in sensory mechanotransduction in proprioceptors. *Nat. Commun.* 7:11460. 10.1038/ncomms11460 27161260 PMC4866049

[B82] Liu Y. Hagan R. Schoellerman J. (2018). Dual actions of Psalmotoxin at ASIC1a and ASIC2a heteromeric channels (ASIC1a/2a). *Sci. Rep.* 8:7179. 10.1038/s41598-018-25386-9 29739981 PMC5940917

[B83] Low C. M. Lyuboslavsky P. French A. Le P. Wyatte K. Thiel W. H. (2003). Molecular determinants of proton-sensitive N-methyl-D-aspartate receptor gating. *Mol. Pharmacol.* 63 1212–1222. 10.1124/mol.63.6.1212 12761330

[B84] Ma B. F. Xie M. J. Zhou M. (2012). Bicarbonate efflux via GABA(A) receptors depolarizes membrane potential and inhibits two-pore domain potassium channels of astrocytes in rat hippocampal slices. *Glia* 60 1761–1772. 10.1002/glia.22395 22855415 PMC3901573

[B85] Ma L. Zhang X. Zhou M. Chen H. (2012). Acid-sensitive TWIK and TASK two-pore domain potassium channels change ion selectivity and become permeable to sodium in extracellular acidification. *J. Biol. Chem.* 287 37145–37153. 10.1074/jbc.M112.398164 22948150 PMC3481314

[B86] MacLean D. M. Jayaraman V. (2016). Acid-sensing ion channels are tuned to follow high-frequency stimuli. *J. Physiol.* 594 2629–2645. 10.1113/JP271915 26931316 PMC4865573

[B87] MacLean D. M. Jayaraman V. (2017). Deactivation kinetics of acid-sensing ion channel 1a are strongly pH-sensitive. *Proc. Natl. Aacd. Sci. U.S.A.* 114 E2504–E2513. 10.1073/pnas.1620508114 28265090 PMC5373395

[B88] Martineau M. Guzman R. E. Fahlke C. Klingauf J. (2017). VGLUT1 functions as a glutamate/proton exchanger with chloride channel activity in hippocampal glutamatergic synapses. *Nat. Commun.* 8:2279. 10.1038/s41467-017-02367-6 29273736 PMC5741633

[B89] Martins D. Tavares I. Morgado C. (2014). “Hotheaded”: the role OF TRPV1 in brain functions. *Neuropharmacology* 85 151–157. 10.1016/j.neuropharm.2014.05.034 24887171

[B90] Mazzuca M. Heurteaux C. Alloui A. Diochot S. Baron A. Voilley N. (2007). Tarantula peptide against pain via ASIC1a channels and opioid mechanisms. *Nat. Neurosci.* 10 943–955. 10.1038/nn1940 17632507

[B91] Mercado F. López I. Ortega A. Almanza A. Soto E. Vega R. (2012). FMRFamide-related peptide expression in the vestibular-afferent neurons. *Neurosci. Lett.* 513 12–16. 10.1016/j.neulet.2012.01.074 22342307

[B92] Mercado F. López I. A. Acuna D. Vega R. Soto E. (2006). Acid-sensing ionic channels in the rat vestibular endorgans and ganglia. *J. Neurophysiol.* 96 1615–1624. 10.1152/jn.00378.2006 16790596

[B93] Mott D. D. Washburn M. S. Zhang S. Dingledine R. J. (2003). Subunit-dependent modulation of kainate receptors by extracellular protons and polyamines. *J. Neurosci.* 23 1179–1188. 10.1523/JNEUROSCI.23-04-01179.2003 12598606 PMC6742282

[B94] Nagaeva E. I. Tikhonova T. B. Magazanik L. G. Tikhonov D. B. (2016). Histamine selectively potentiates acid-sensing ion channel 1a. *Neurosci. Lett.* 632 136–140. 10.1016/j.neulet.2016.08.047 27574729

[B95] N’Gouemo P. (2008). Amiloride delays the onset of pilocarpine-induced seizures in rats. *Brain Res.* 1222 230–232. 10.1016/j.brainres.2008.05.010 18572151 PMC2562261

[B96] Obara M. Szeliga M. Albrecht J. (2008). Regulation of pH in the mammalian central nervous system under normal and pathological conditions: facts and hypotheses. *Neurochem. Int.* 52 905–919. 10.1016/j.neuint.2007.10.015 18061308

[B97] Ortega-Ramírez A. Vega R. Soto E. (2017). Acid-sensing ion channels as potential therapeutic targets in neurodegeneration and neuroinflammation. *Mediators Inflamm.* 2017:3728096. 10.1155/2017/3728096 29056828 PMC5625748

[B98] Osmakov D. I. Andreev Y. A. Kozlov S. A. (2014). Acid sensing ion channels and their modulators. *Biochemistry* 79 1528–1545. 10.1134/S0006297914130069 25749163

[B99] Osmakov D. I. Koshelev S. G. Andreev Y. A. Dubinnyi M. A. Kublitski V. S. Efremov R. G. (2018). Proton-independent activation of acid-sensing ion channel 3 by an alkaloid, lindoldhamine, from *Laurus nobilis*. *Br. J. Pharmacol.* 175 924–937. 10.1111/bph.14134 29277899 PMC5825300

[B100] Parker M. D. Boron W. F. (2013). The divergence, actions, roles, and relatives of sodium-coupled bicarbonate transporters. *Physiol. Rev.* 93 803–959. 10.1152/physrev.00023.2012 23589833 PMC3768104

[B101] Peng B. G. Ahmad S. Chen S. Chen P. Price M. P. Lin X. (2004). Acid-sensing ion channel 2 contributes a major component to acid-evoked excitatory responses in spiral ganglion neurons and plays a role in noise susceptibility of mice. *J. Neurosci.* 24 10167–10175. 10.1523/JNEUROSCI.3196-04.2004 15537887 PMC6730178

[B102] Pfeiffer J. Johnson D. Nehrke K. (2008). Oscillatory transepithelial H^+^ flux regulates a rhythmic behavior in *C. elegans*. *Curr. Biol.* 18 297–302. 10.1016/j.cub.2008.01.054 18291648 PMC2350219

[B103] Pignataro G. Simon R. P. Xiong Z. G. (2007). Prolonged activation of ASIC1a and the time window for neuroprotection in cerebral ischaemia. *Brain* 130 151–158. 10.1093/brain/awl325 17114797

[B104] Ramirez S. Allen T. Villagracia L. Chae Y. Ramirez J. M. Rubens D. D. (2016). Inner ear lesion and the differential roles of hypoxia and hypercarbia in triggering active movements: potential implication for the Sudden Infant Death Syndrome. *Neuroscience* 337 9–16. 10.1016/j.neuroscience.2016.08.054 27634772

[B105] Rothschild L. J. Mancinelli R. L. (2001). Life in extreme environments. *Nature* 409 1092–1101. 10.1038/35059215 11234023

[B106] Ruffin V. A. Salameh A. I. Boron W. F. Parker M. D. (2014). Intracellular pH regulation by acid-base transporters in mammalian neurons. *Front. Physiol.* 5:43 10.3389/fphys.2014.00043 PMC3923155 24592239

[B107] Schmidt A. Lenzig P. Oslender-Bujotzek A. Kusch J. Lucas S. D. Gründer S. (2014). The bile acid-sensitive ion channel (BASIC) is activated by alterations of its membrane environment. *PLoS One* 9:e111549. 10.1371/journal.pone.0111549 25360526 PMC4216111

[B108] Sherwood T. W. Lee K. G. Gormley M. G. Askwith C. C. (2011). Heteromeric acid-sensing ion channels (ASICs) composed of ASIC2b and ASIC1a display novel channel properties and contribute to acidosis-induced neuronal death. *J. Neurosci.* 31 9723–9734. 10.1523/JNEUROSCI.1665-11.2011 21715637 PMC3160670

[B109] Sinning A. Hübner C. A. (2013). Minireview: pH and synaptic transmission. *FEBS Lett.* 587 1923–1928. 10.1016/j.febslet.2013.04.045 23669358

[B110] Slepkov E. R. Rainey J. K. Sykes B. D. Fliegel L. (2007). Structural and functional analysis of the Na^+^/H^+^ exchanger. *Biochem. J.* 401 623–633. 10.1042/BJ20061062 17209804 PMC1770851

[B111] Sluka K. A. Price M. P. Breese N. M. Stucky C. L. Wemmie J. A. Welsh M. J. (2003). Chronic hyperalgesia induced by repeated acid injections in muscle is abolished by the loss of ASIC3, but not ASIC1. *Pain* 106 229–239. 10.1016/S0304-3959(03)00269-0 14659506

[B112] Sluka K. A. Radhakrishnan R. Benson C. J. Eshcol J. O. Price M. P. Babinski K. (2007). ASIC3 in muscle mediates mechanical, but not heat, hyperalgesia associated with muscle inflammation. *Pain* 129 102–112. 10.1016/j.pain.2006.09.038 17134831 PMC1941845

[B113] Soto E. Vega R. González A. Ortega A. (2014). “Acid sensing ionic channels mediate an excitatory synaptic input to the cochlear and vestibular afferent neurons,” in *Proceedings of the 37th Annual MidWinter Meeting of the Association for Research in Otolaryngology*, San Diego, CA.

[B114] Storozhuk M. Kondratskaya E. Nikolaenko L. Krishtal O. (2016). A modulatory role of ASICs on GABAergic synapses in rat hippocampal cell cultures. *Mol. Brain* 9:90. 10.1186/s13041-016-0269-4 27760555 PMC5070181

[B115] Suman A. Mehta B. Guo M. L. Chu X. P. Fibuch E. E. Mao L. M. (2010). Alterations in subcellular expression of acid-sensing ion channels in the rat forebrain following chronic amphetamine administration. *Neurosci. Res.* 68 1–8. 10.1016/j.neures.2010.06.001 20566346 PMC2917493

[B116] Tian Y. H. Lee S. Y. Kim H. C. Jang C. G. (2010). Repeated methamphetamine treatment increases expression of TRPV1 mRNA in the frontal cortex but not in the striatum or hippocampus of mice. *Neurosci. Lett.* 472 61–64. 10.1016/j.neulet.2010.01.058 20122992

[B117] Tian Y. H. Ma S. X. Lee K. W. Wee S. Koob G. F. Lee S. Y. (2018). Blockade of TRPV1 inhibits methamphetamine-induced rewarding effects. *Sci. Rep.* 8:882. 10.1038/s41598-018-19207-2 29343767 PMC5772440

[B118] Tombaugh G. C. Somjen G. G. (1996). Effects of extracellular pH on voltage-gated Na^+^, K^+^ and Ca2_+_ currents in isolated rat CA1 neurons. *J. Physiol.* 493 719–732. 10.1113/jphysiol.1996.sp021417 8799894 PMC1159020

[B119] Toth A. Boczan J. Kedei N. Lizanecz E. Bagi Z. Papp Z. (2005). Expression and distribution of vanilloid receptor 1 (TRPV1) in the adult rat brain. *Brain Res. Mol. Brain Res.* 135 162–168. 10.1016/j.molbrainres.2004.12.003 15857679

[B120] Traynelis S. F. Cull-Candy S. G. (1990). Proton inhibition of N-methyl- D-aspartate receptors in cerebellar neurons. *Nature* 345 347–350. 10.1038/345347a0 1692970

[B121] Traynelis S. F. Wollmuth L. P. McBain C. J. Menniti F. S. Vance K. M. Ogden K. K. (2010). Glutamate receptor ion channels: structure, regulation, and function. *Pharmacol. Rev.* 62 405–496. 10.1124/pr.109.002451 20716669 PMC2964903

[B122] Ugawa S. Inagaki A. Yamamura H. Ueda T. Ishida Y. Kajita K. (2006). Acid-sensing ion cannel-Ib in the stereocilia of mammalian cochlear hair cells. *Neuroreport* 17 1235–1239. 10.1097/01.wnr.0000233093.67289.66 16951561

[B123] Vega R. Rodriguez U. Soto E. (2009). Acid-sensing ionic-channel functional expression in the vestibular endorgans. *Neurosci. Lett.* 463 199–202. 10.1016/j.neulet.2009.07.086 19660522

[B124] Verma V. Bali A. Singh N. Jaggi A. S. (2015). Implications of sodium hydrogen exchangers in various brain diseases. *J. Basic Clin. Physiol. Pharmacol.* 26 417–426. 10.1515/jbcpp-2014-0117 26020555

[B125] Vullo S. Bonifacio G. Roy S. Johner N. Bernèche S. Kellenberger S. (2017). Conformational dynamics and role of the acidic pocket in ASIC pH-dependent gating. *Proc. Natl. Acad. Sci. U.S.A.* 114 3768–3773. 10.1073/pnas.1620560114 28320963 PMC5389290

[B126] Walder R. Y. Gautam M. Wilson S. P. Benson C. J. Sluka K. A. (2011). Selective targeting of ASIC3 using miRNAs inhibits primary and secondary hyperalgesia following muscle inflammation. *Pain* 152 2348–2356. 10.1016/j.pain.2011.06.027 21843914 PMC3476729

[B127] Waldmann R. Champigny G. Bassilana F. Heurteaux C. Lazdunski M. (1997). A proton-gated cation channel involved in acid-sensing. *Nature* 386 173–177. 10.1038/386173a0 9062189

[B128] Wang T. M. Holzhausen L. C. Kramer R. H. (2014). Imaging an optogenetic pH sensor reveals that protons mediate lateral inhibition in the retina. *Nat. Neurosci.* 17 262–268. 10.1038/nn.3627 24441679 PMC3985427

[B129] Wang Y. O’Bryant Z. Wang H. Huang Y. (2016). Regulating factors in acid-sensing ion channel 1a function. *Neurochem. Res.* 41 631–645. 10.1007/s11064-015-1768-x 26582234

[B130] Warren T. J. Van Hook M. J. Supuran C. T. Thoreson W. B. (2016). Sources of protons and a role for bicarbonate in inhibitory feedback from horizontal cells to cones in *Ambystoma tigrinum* retina. *J. Physiol.* 594 6661–6677. 10.1113/JP272533 27345444 PMC5108903

[B131] Wemmie J. A. Askwith C. C. Lamani E. Cassell M. D. Freeman J. H. Jr. Welsh M. J. (2003). Acid-sensing ion channel 1 is localized in brain regions with high synaptic density and contributes to fear conditioning. *J. Neurosci.* 23 5496–5502. 10.1523/JNEUROSCI.23-13-05496.2003 12843249 PMC6741257

[B132] Wemmie J. A. Taugher R. J. Kreple C. J. (2013). Acid-sensing ion channels in pain and disease. *Nat. Rev. Neurosci.* 14 461–471. 10.1038/nrn3529 23783197 PMC4307015

[B133] Weng X. C. Zheng J. Q. Jin Q. E. Ma X. Y. (2007). Inhibition of acid-induced apoptosis by targeting ASIC1a mRNA with short hairpin RNA. *Acta Pharmacol. Sin.* 28 1621–1627. 10.1111/j.1745-7254.2007.00627.x 17883949

[B134] Wiemann M. Frede S. Tschentscher F. Kiwull-Schöne H. Kiwull P. Bingmann D. (2008). NHE3 in the human brainstem: implication for the pathogenesis of the sudden infant death syndrome (SIDS)? *Adv. Exp. Med. Biol.* 605 508–513. 10.1007/978-0-387-73693-8_89 18085326

[B135] Wu H. Wang C. Liu B. Li H. Zhang Y. Dong S. (2016). Altered expression pattern of acid-sensing ion channel isoforms in piriform cortex after seizures. *Mol. Neurobiol.* 53 1782–1793. 10.1007/s12035-015-9130-5 25744567

[B136] Wu J. Xu Y. Jiang Y. Q. Xu J. Hu Y. Zha X. M. (2016). ASIC subunit ratio and differential surface trafficking in the brain. *Mol. Brain* 9:4. 10.1186/s13041-016-0185-7 26746198 PMC4706662

[B137] Xiong Z. G. Pignataro G. Li M. Chang S. Y. Simon R. P. (2008). Acid-sensing ion channels (ASICs) as pharmacological targets for neurodegenerative diseases. *Curr. Opin. Pharmacol.* 8 25–32. 10.1016/j.coph.2007.09.001 17945532 PMC2267925

[B138] Yang L. Faraone S. V. Zhang-James Y. (2016). Autism spectrum disorder traits in Slc9a9 knock-out mice. *Am. J. Med. Genet. Part B* 171B, 363–376. 10.1002/ajmg.b.32415 26755066

[B139] Yang L. Palmer L. G. (2014). Ion conduction and selectivity in acid-sensing ion channel 1. *J. Gen. Physiol.* 144 245–255. 10.1085/jgp.201411220 25114023 PMC4144671

[B140] Yermolaieva O. Leonard A. S. Schnizler M. K. Abboud F. M. Welsh M. J. (2004). Extracellular acidosis increases neuronal cell calcium by activating acid-sensing ion channel 1a. *Proc. Natl. Acad. Sci. U.S.A.* 101 6752–6757. 10.1073/pnas.0308636100 15082829 PMC404117

[B141] Yoder N. Yoshioka C. Gouaux E. (2018). Gating mechanisms of acid-sensing ion channels. *Nature* 555 397–401. 10.1038/nature25782 29513651 PMC5966032

[B142] You I. J. Jung Y. H. Kim M. J. Kwon S. H. Hong S. I. Lee S. Y. (2012). Alterations in the emotional and memory behavioral phenotypes of transient receptor potential vanilloid type 1-deficient mice are mediated by changes in expression of 5-HT1A, GABAA, and NMDA receptors. *Neuropharmacology* 62 1034–1043. 10.1016/j.neuropharm.2011.10.013 22074644

[B143] Yu Y. Chen Z. Li W. G. Cao H. Feng E. G. Yu F. (2010). A nonproton ligand sensor in the acid-sensing ion channel. *Neuron* 68 61–72. 10.1016/j.neuron.2010.09.001 20920791

[B144] Zeng W. Z. Liu D. S. Liu L. She L. Wu L. J. Xu T. L. (2015). Activation of acid-sensing ion channels by localized proton transient reveals their role in proton signaling. *Sci. Rep.* 5:14125. 10.1038/srep14125 26370138 PMC4569896

[B145] Zha X. M. Costa V. Harding A. M. Reznikov L. Benson C. J. Welsh M. J. (2009). ASIC2 subunits target acid-sensing ion channels to the synapse via an association with PSD-95. *J. Neurosci.* 29 8438–8446. 10.1523/JNEUROSCI.1284-09.2009 19571134 PMC2734339

[B146] Zhang H. Gao G. Zhang Y. Sun Y. Li H. Dong S. (2017). Glucose deficiency elevates acid-sensing ion channel 2a expression and increases seizure susceptibility in temporal lobe epilepsy. *Sci. Rep.* 7:5870. 10.1038/s41598-017-05038-0 28725010 PMC5517604

[B147] Zhang G.-C. Mao L.-M. Wang J. Q. Chu X.-P. (2009). Upregulation of acid-sensing ion channel 1 protein expression by chronic administration of cocaine in the mouse striatum *in vivo*. *Neurosci. Lett.* 459 119–122. 10.1016/j.neulet.2009.05.013 19427358 PMC2737317

[B148] Zhang P. Canessa C. M. (2002). Single channel properties of rat acid–sensitive ion channel-1α, -2a, and -3 expressed in *Xenopus* oocytes. *J. Gen. Physiol.* 120 553–566. 10.1085/jgp.20028574 12356856 PMC2229538

[B149] Zhao D. Ning N. Lei Z. Sun H. Wei C. Chen D. (2014). Identification of a novel protein complex containing ASIC1a and GABAA receptors and their interregulation. *PLoS One* 9:e99735. 10.1371/journal.pone.0099735 24923912 PMC4055689

[B150] Zhao H. Carney K. E. Falgoust L. Pan J. W. Sun D. Zhang Z. (2016). Emerging roles of Na^+^/H^+^ exchangers in epilepsy and developmental brain disorders. *Prog. Neurobiol.* 138 19–35. 10.1016/j.pneurobio.2016.02.002 26965387 PMC4852136

[B151] Ziemann A. E. Allen J. E. Dahdaleh N. S. Drebot I. I. Coryell M. W. Wunsch A. M. (2009). The amygdala is a chemosensor that detects carbon dioxide and acidosis to elicit fear behavior. *Cell* 139 1012–1021. 10.1016/j.cell.2009.10.029 19945383 PMC2808123

[B152] Ziemann A. E. Schnizler M. K. Albert G. W. Severson M. A. Howard M. A. III Welsh M. J. (2008). Seizure termination by acidosis depends on ASIC1a. *Nat. Neurosci.* 11 816–822. 10.1038/nn.2132 18536711 PMC2553357

[B153] Zuo Z. Smith R. N. Chen Z. Agharkar A. S. Snell H. D. Huang R. (2018). Identification of a unique Ca2^+^-binding site in rat acid-sensing ion channel 3. *Nat. Commun.* 9:2082. 10.1038/s41467-018-04424-0 29802295 PMC5970173

